# Illuminating Immunity: A Systematic Review of Immune Cell Autofluorescence

**DOI:** 10.1002/jbio.202400576

**Published:** 2025-03-20

**Authors:** Aline Knab, Caroline Giardina, Shane T. Grey, Ewa M. Goldys, Jared M. Campbell

**Affiliations:** ^1^ Graduate School of Biomedical Engineering University of New South Wales Sydney New South Wales Australia; ^2^ Garvan Institute of Medical Research Darlinghurst New South Wales Australia; ^3^ Faculty of Medicine & Health University of New South Wales Sydney New South Wales Australia; ^4^ School of Biotechnology and Biomolecular Sciences University of New South Wales Sydney New South Wales Australia

**Keywords:** autofluorescence, immune cells, immunophenotyping, label free

## Abstract

Immunophenotyping provides valuable prognostic and diagnostic information, but is technically complex and expensive. The assessment of autofluorescence is label‐free and provides complex information on cell identity. However, research on its application to immunophenotyping has been heterogenous. This systematic review was carried out to identify and synthesise all available evidence on the use of autofluorescence for immunophenotyping. Eighty three full texts were included. There was a focus on neutrophils (20 papers) and macrophages (22 papers) with alveolar macrophages (13 papers) forming a subcategory. Seven studies investigated monocytes, three focused on microglia, two on dendritic cells, five on mast cells, nine on granulocytes, thirteen on eosinophils, one on erythrophagocytic cells, and one on natural killer cells. Eleven studies investigated uncategorised immune cell populations. Translation of findings into clinical immunophenotyping requires the application of reproducible methods, along with clear reporting of excitation and emission parameters, and a greater focus on clinical and primary samples.

## 1 Introduction

The immune system forms a highly diverse and intricate network, comprising numerous cell types that exhibit specialised functions and dynamic activation states. The immune system relies on a diversity of immune cells, each contributing to the body's defense through innate and adaptive immunity. Innate immunity provides the first line of defense through rapid, non‐specific responses, primarily involving macrophages, neutrophils, and dendritic cells, while adaptive immunity, driven by lymphocytes such as T cells and B cells, offers highly specific, long‐lasting protection through antigen recognition and immune memory [1]. The evaluation of immune cell populations in biomedical research and clinical medicine (e.g., in the diagnosis of haematological malignancies such as lymphomas and leukemia) is generally carried out by immunophenotyping. A comprehensive characterisation of immune cell phenotypes requires the detection of numerous biomarkers using multiple fluorescent antibody staining, often requiring up to 50 or more antibodies [2]. This protocol can be technically demanding, time‐intensive, and costly. Given the bidirectional relationship between health and the immune system, immunophenotyping provides valuable diagnostic and prognostic [3] insights in a range of diseases, which can aid clinical decision‐making. For example, a recent study [4] evaluated 650 cell types and their activation states in healthy donors and patients with advanced cancers using multiple panels of markers. Unsupervised clustering revealed five general immunophenotypes, one of which was associated with predicting immunotherapy treatment response in head and neck squamous cell carcinoma patients. In another work, immunophenotyping was used to characterise autoimmune and autoinflammatory disorders, demonstrating two distinctive types of immunological responses and suggesting a novel treatment paradigm based on immune cell parameters [5]. Further challenges to conventional immunophenotyping methods include variability in sample preparation and processing, which can affect the reproducibility and transferability of results. Additionally, there is often disagreement on which markers are necessary. Another limitation is the destructive nature of certain assays, which prevents their use in longitudinal studies and combinatorial experiments with other assessment methods, a challenge particularly pronounced in scenarios with limited sample availability such as neonatal blood. Furthermore, reductionist approaches focused on narrow ranges of biomarkers and specific cell types have been identified as a barrier to establishing robust models for immunophenotyping [4]. As such, an inexpensive, label‐free methodology for comprehensive immunophenotyping would be an invaluable tool for research and healthcare.

**FIGURE 1:**
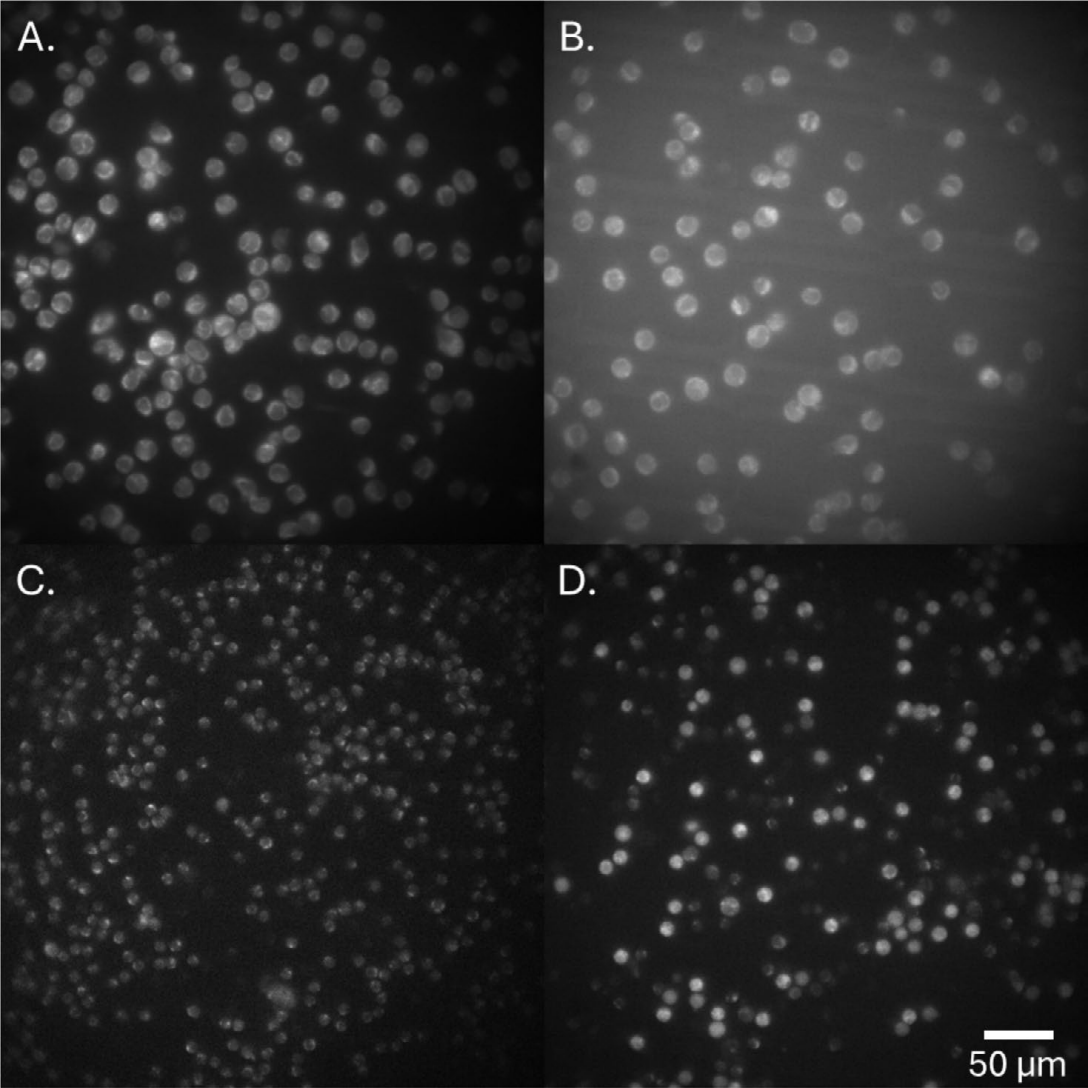
Single photon 40× fluorescence microscopy of (A) THP‐1 monocytes, (B) THP‐1 monocytes partially differentiated into macrophages, (C) B cells, (D) uncategorised peripheral blood mono nuclear cells, excited at 356 ± 5 nm with emissions captured from 396 to 437 nm, primarily representing NAD(P)H autofluorescence.

**FIGURE 2:**
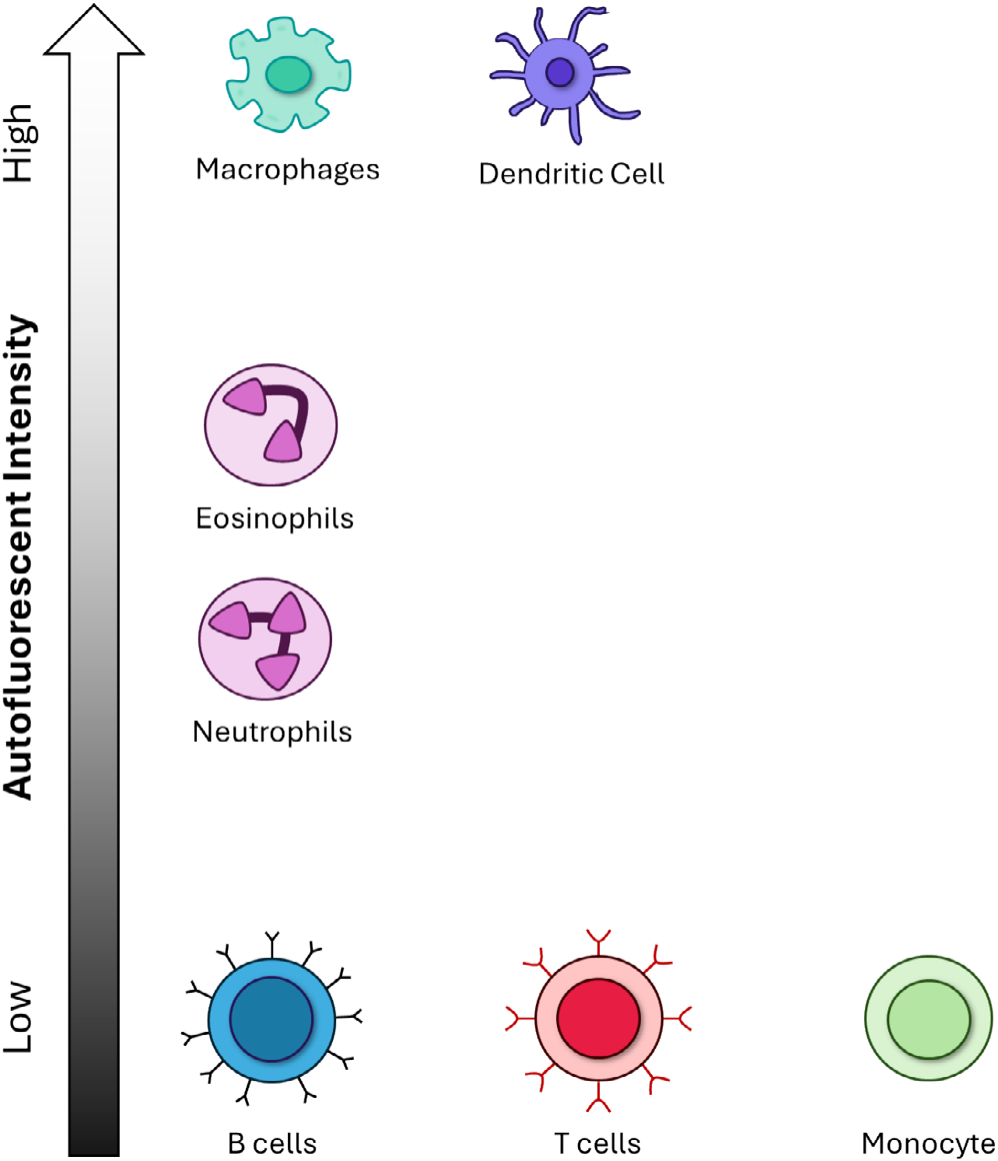
General autofluorescent intensities of major immune cell types. Macrophages and dendritic cells exhibit high autofluorescence [54, 56], while eosinophils show higher autofluorescence levels compared to neutrophils [43, 44, 46, 105, 106, 107, 114, 145]. B cells, T cells, and monocytes display low autofluorescence intensities [43, 44, 46, 54, 56, 76].

One alternative approach to immunophenotyping is the assessment of immune cell autofluorescence [6]. Numerous intracellular molecules are naturally fluorescent with distinct spectral profiles. Example autofluorophores include the principal electron donors and acceptors of oxidative phosphorylation NAD(P)H (Figure 1) and FAD, the mitochondrial inner membrane protein cytochrome c, the lysosomal waste product lipofuscin, and the structural collagen proteins [7, 8]. Due to their functional contributions, and differing intensities (Figure 2) the spectral assessment of cellular autofluorescence is a powerful tool for the identification of cell identity and status. Successful applications of autofluorescence assessment have included tumour surgical margin definition [9, 10, 11], assessment of the impact of age and drug exposure on stem cells [12, 13], and cell specific properties such as levels of ROS and cell cycle stage [14, 15, 16].

Autofluorophores can be excited by single photons or by multiphoton (typically two‐photon) technology. For multiphoton excitation, the energy of several photons is combined to excite an autofluorophore's electron (the wavelength of the exciting photons is proportionally increased due to the lower energy state needed). Subsequently the electron shifts back to its initial state, producing an emission. Multiphoton excitation events are much less probable than single photon events as they rely on simultaneous absorption of two or more photons. Consequently, multiphoton microscopy relies on higher intensity light sources to achieve detectable signal. Single photon fluorescence can be excited by comparatively weak and inexpensive light sources, however, the short wavelengths of light used to induce fluorescence result in lower tissue penetration.

The two main methodologies for assessing autofluorescence in cells and tissues include flow cytometry and microscopy (single, multiphoton and fluorescence lifetime imaging microscopy (FLIM)—which measures time decay information enabling the discrimination of otherwise spectrally identical fluorophores). Microscopy has the advantage of capturing morphological information pertaining to cell structure and organisation which can add important information for cell type identification and assessment [17], but the number of imaged cells is comparatively limited and assessment speed is low. In contrast, flow cytometry allows for high‐throughput analysis of large cell populations, making it invaluable for immunophenotyping, which often requires the assessment of hundreds of cell types and activation states [4, 5].

Studies of the autofluorescent properties of immune cells and their application in identification and characterisation have been conducted for decades [18, 19]. However, published study designs are heterogenous and progress towards practical applications has been slow. As such, we have undertaken this systematic review to identify and synthesise all available evidence on the use of autofluorescence for the assessment and characterisation of immune cells. Our goal is for this work to provide a foundation that supports the translation and implementation of this promising technology into both research and clinical practice.

## 2 Methods

**FIGURE 3:**
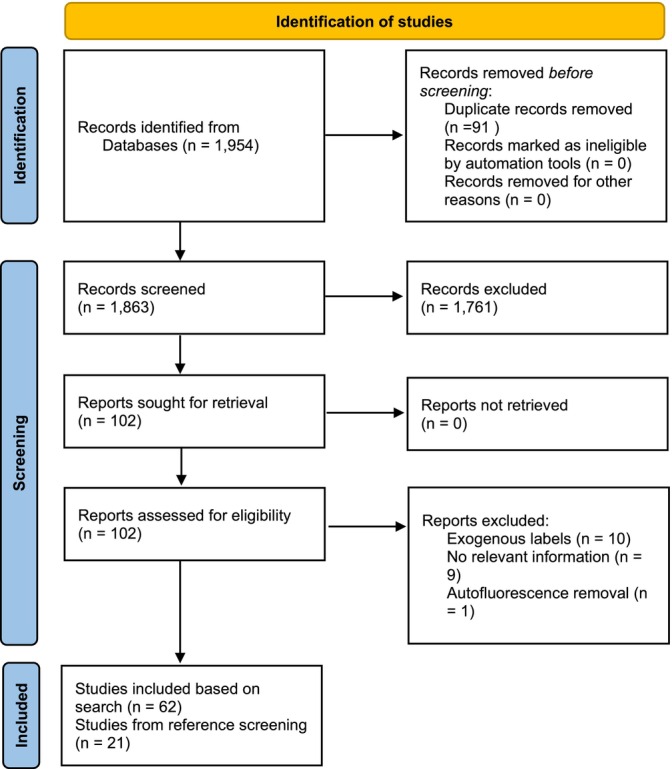
PRISMA 2020 flow diagram [150].

This systematic review was carried out according to a pre‐defined protocol (Figure 3) to identify and synthesise all available evidence on the use of autofluorescence for assessing and characterising immune cells.

### 2.1 Inclusion Criteria

Studies were eligible for inclusion where they utilised autofluorescence (excited and captured by any technology, for example, single photon, multiphoton fluorescence lifetime imaging microscopy, flow cytometry) for the identification or characterisation of immune cells. Any form of immune cell was eligible for inclusion, including tissue resident and circulating blood immune cells. The collection of autofluorescence data had to be separable from exogenous staining or transformation for inclusion (e.g., a study would have been eligible for inclusion if labelling with a far red fluorophore was used for the identification or isolation of an immune cell, followed by characterisation of its autofluorescence in the UV range, but not a study where a combination of exogenous markers and autofluorescence were used for the prediction of cell type). All publication types were eligible for inclusion, including grey literature and pre‐prints. No date restrictions were applied, however studies in languages other than English were not assessed. Study designs included in this review encompass human and murine primary cells, cell lines and animal models.

### 2.2 Search Strategy

The search strategy (carried out in September 2023) aimed to find both published and unpublished studies. An initial limited search of Medline was undertaken followed by an analysis of text words contained in titles and abstracts, as well as the index terms used to describe the articles. A second search using all identified terms was then undertaken across Medline (using the Pubmed portal) and Google Scholar. The search strategy included search terms related to immune cells (e.g., T cells, neutrophils, peripheral blood mononuclear cell (PBMC), immune, immunology) and autofluorescence (e.g., autofluorophore, endogenous fluorescence, native fluorescence).

### 2.3 Data Extraction and Narrative Synthesis

Data were extracted using a pre‐defined ruberic with fields for study aim, measurement technique, test object, stimulation protocol, evaluation technique, accuracy, and results. Due to the heterogeneity of study designs and contexts no meta‐analysis has been undertaken. Instead, a narrative synthesis approach focused on cell type, characterisation, the impact of pathological statuses and identification has been used to pool findings by common elements [20].

## 3 Results

On PubMed the search strategy resulted in 954 potential studies (Figure 3). On Google Scholar there were 41 700 items, from which the top 1000 sorted by “relevance” (the maximum permitted by the system) were retrieved. These 1954 studies were reduced to 1863 by the removal of duplication. Screening of titles and abstracts resulted in the identification of 102 studies for full text retrieval and review, from these 62 met the inclusion criteria. Reasons for exclusion included the use of exogenous labels [21, 22, 23, 24, 25, 26, 27, 28, 29, 30], lack of relevant data [31, 32, 33, 34, 35, 36, 37, 38, 39], and in one instance, the study of autofluorescence signal removal [40]. The references of the selected papers were screened, leading to the identification of 21 additional studies that met the inclusion criteria.

In the 84 included studies, a significant focus was placed on neutrophils (20 papers) and macrophages (22 papers) with alveolar macrophages (13 papers) forming a subcategory. Additionally, seven studies examined monocytes, three assessed microglia, two dendritic cells, five mast cells, 9 granulocytes, 13 eosinophils, one erythrophagocytic cells, and one natural killer cells. Overall, cells from the innate immune system were investigated in 65 studies (Table 1), while cells from the adaptive immune system were investigated in 24 studies (Table 2). Uncategorised lymphocytes were addressed in 11 studies, 13 provided information on T cells and four on B cells. Several studies covered information on more than one immune cell type, while 10 studies reported uncategorised immune cell autofluorescence without specifying or differentiating the cell type (Table 3) (e.g., the characterisation of unsorted peripheral blood mononuclear cells, PBMCs).

**TABLE 1:** Overview on innate immunity and their autofluorescent properties (WL: wavelength, ex: excitation, em: emission, AF: autofluorescence, ^2^adaptive immune cells, ^3^uncategorised immune cells).

Paper	Cell type	System	Assessment	WL for AF	Aim/conclusion
[47]	Monocytes, macrophages (RAW264.7)	Human, cell line	Metabolic oscillations (NAD(P)H)	ex: 340–390 nm; em: 421–479 nm	NAD(P)H AF exhibited oscillations with ~20 s periods, enhanced by IFN‐γ and CpG DNA activation.
[61]	Macrophages	Mouse	FLIM, two‐photon excitation	ex: 740 nm, em: 420–500 nm	Bone marrow‐derived macrophages showed higher AF from bound NADH, indicating increased glycolytic metabolism.
[105]	Neutrophils, eosinophils	Human	Spectrofluorimetry	ex: 250–500 nm with 10 nm increment, em: ex +10 nm to [2 × ex—10 nm or 700 nm, 5 nm increment]	Neutrophils had weak AF in contrast to eosinophils for which the color of AF differed for isolated cells and tissue cells.
[84]	Alveolar macrophages, granulocytes	Pig	Flow cytometry	ex: 488 nm, em: green & orange fluorescence	Macrophages in healthy and infected animals were identified by their high green AF, with more highly autofluorescent cells present in diseased animals.
[59]	Macrophages	Mouse, human	Flow cytometry	SP6800 Spectral Cell Analyzer (SONY Biotechnology)	Autofluorescent macrophages had higher IL‐6 and TNF‐α production upon stimulation and represented a highly phagocytic subtype in cancerous skin tissue compared to non‐autofluorescent ones.
[92]	Mast cells, neutrophils, eosinophils	Human (lymphoma tissue)	Fluorescence imaging	ex: 450–490 nm, em: 500–550 nm; ex: 535–555 nm, em: 575–635 nm; ex: 625–655 nm, em: 665–715 nm	Mast cells and neutrophils did not display observable AF in contrast to eosinophils.
[74]	Alveolar macrophages	Human	Laser scanning cytometry	ex: 488 nm, 633 nm; em: not defined	Weak and variable AF in fixed pediatric BAL samples limited the detection of individual alveolar macrophages, only identifying cell clumps.
[89]	Microglia	Mouse, non‐human primate	Flow cytometry, FACS	5 laser‐equipped LSRFortessa X‐20, BD FACSAria Fusion	Two microglial subsets differentiated by AF were found, with increased lysosomal complexity and elevated ROS production in the autofluorescent subset, suggesting a role in oxidative stress and aging. AF levels increased with age.
[82]	Alveolar macrophages, neutrophils, leukocytes^3^	Mouse (lymph nodes, BAL, lung tissue)	Flow cytometry	Cytek Aurora 5	Adjusting for the AF signals from alveolar macrophages and neutrophils enhanced the accuracy of unmixing techniques and gating strategies in flow cytometry.
[68]	Macrophages	Mouse	FLIM, two‐photon excitation	ex: 740 nm, em: 400–480, (NAD(P)H); ex: 890 nm, em: 510–600 nm (FAD); ex: 1040 nm, em: 604–679 nm (lipofuscin)	Investigation of prediabetes and diabetes in mouse adipose tissue revealed lipofuscin‐like AF specific to macrophages, effectively distinguishing them from adipocytes.
[106]	Neutrophils. eosinophils	Human, mouse	FACS	ex: 355 nm, em: 425–475 nm; ex: 488 nm, em: 500–550 nm; ex: 488 nm, em: 484–492 nm (SCC); ex: 561 nm, em: 577.5–592.5 nm	Weak AF in neutrophils contrasted with strong AF in eosinophils, facilitating highly pure cell separation through FACS.
[49]	Monocytes	Human	Microspectro‐fluorometry	ex: 355 nm, em: 300–700 nm	In individuals with elevated cholesterol levels, human monocytes displayed significantly increased AF.
[18]	Neutrophils, eosinophils	Human	Fluorescence microscopy	ex: 335–480 nm, em: > 525 nm	Neutrophils displayed faint yellow‐green AF, while eosinophil granules exhibited intense yellow AF, with no clear correlation to disease or therapy.
[56]	Macrophages, dendritic cells, B cells^2^, T cells^2^	Mouse	FLIM, two‐photon excitation	ex in vitro: 710 to 830 nm, ex in vivo: 710 to 750 nm, em: 350 to 600 nm	Macrophages exhibited higher AF and different lifetime characteristics based on excitation wavelength, especially under PMA activation and in inflamed tissue. AF patterns of macrophages, dentritic cells, B cells and T cells differed with macrophages emitting the highest intensities.
			FLIM, two‐photon excitation	ex: 710, 730, 750, 780, 800 and 830 nm, em: 380–450 nm, 450–500 nm, 500–580 nm, 580–680 nm	
[48]	Monocytes, macrophages (P388D1)	Human, cell line	Laser‐induced fluorescence spectroscopy	ex: 308 nm, em: ~315–670 nm	Monocytes from individuals with elevated cholesterol showed higher AF due to oxidised LDL and macrophages incubated with oxidised low‐density lipoprotein (LDL) were found to take on its spectral characteristics.
[110]	Neutrophils	Human	Flow cytometry	ex: 350 nm, em: > 425 nm	AF in neutrophils was linked to NAD(P)H, with changes observed post‐inhibition of glucose‐dependent pyridine nucleotide reduction and decreased AF upon activation.
[50]	Monocytes, macrophages	Cell line (human, mouse)	FLIM, two‐photon excitation	ex: 750 nm, em: 446–486 nm (NAD(P)H); ex: 890 nm, em: 528–552 (FAD)	Monocytes co‐cultured with breast cancer cells developed AF patterns akin to macrophages, with dynamic changes in NAD(P)H and FAD over time reflecting metabolic heterogeneity and cell migration.
[63]	Macrophages	Mouse	FLIM, two‐photon excitation	ex: 750 nm, em: 446–486 nm (NAD(P)H); ex: 750 nm, em: 499–529 nm FAD	Dermal macrophages had longer NAD(P)H and FAD fluorescence lifetimes, indicating metabolic differences from tumor macrophages, with greater heterogeneity in optical redox ratios.
[101]	Polymorphonuclear cells	Human	Fluorescence spectroscopy	ex: 250–550 nm, 10 nm increments em: up to 700 nm, 5 nm increments ex: 290 nm, em: 330 nm (tryptophan); ex: 350 nm, em: 450 nm (NAD(P)H); ex: 450 nm, em: 530 nm (FAD); ex: 500 nm, em: 530 nm (unidentified)	EEM revealed tryptophan, NAD(P)H, and FAD AF for leukocytes, with increased tryptophan upon fMLP stimulation.
[72]	Macrophages (RAW264.7)	Cell line (Mouse)	Flow cytometry	ex: 350 nm, 488, 561 nm, 638, 808 nm; em: 485–491 nm (SSC), 427.5–472.5 nm, 505–545 nm, 564–606 nm, 600–620 nm, 660–690 nm, 665–715 nm, 685–735 nm, 699.5–724.5 nm, 741.5–784.5 nm, 830–850 nm, 865–905 nm (not all ex/em combinations considered)	Six macrophage phenotypes were discriminated with high accuracies, with the lowest confusion between M0, M2b, M1 and M2d macrophages, and highest between M0, M2c and M2a which have similar gene and protein expression.
[85]	Alveolar macrophages, lymphocytes^2^, leukocytes^3^	Mouse, human	Flow cytometry	CytoFLEX LX, Cytek Aurora 5 L	Fixed blood lymphocytes exhibited higher AF levels than live ones for UV, violet, and blue excitation in spectral flow cytometry
[57]	Macrophages (RAW264.7)	Cell line	Fluorescence microscopy, two‐photon excitation	ex: 710 nm, em: 400–560 nm	Exposure to glucose or NaCN increased NAD(P)H AF, while activators of NADPH oxidase decreased it.
[77]	Alveolar macrophages	Human	Fluorescence microscopy	ex: 450–490 nm, em: > 515 nm	Alveolar macrophages in smokers displayed red‐brown cytoplasmic AF, independent of particle deposits, varying with biopsy location and patient age.
[103]	Granulocytes, lymphocytes^2^	Mouse	Fluorescence microscopy, two‐photon excitation	ex: (1) 800 nm (bandwidth > 150 nm), (2) 730–800 nm (tuneable); em: 396–452 nm, 446–499 nm, 499–582 nm, 583–671 nm	AF distinguished granulocytes and non‐AF lymphocytes.
[113]	Neutrophils, eosinophils	Mouse	Fluorescence microscopy, two‐photon excitation	ex: 750 nm (tissue AF), 800 nm (collagen); em: 380–450 nm, 450–500 nm, 500–580 nm, 580–680 nm	Neutrophils and eosinophils could not be distinguished based on AF.
[55]	Macrophages (A3.1A), microglia (MyD88/TRIF double KO)	Mouse cell line	FACS	ex: 488 nm, em: 518–548 nm, 565–605 nm	Demonstrated that feeding microglia and macrophages with oxidised rod outer segment increases lipofuscin‐like AF, supporting the role of oxidative stress in lipofuscinogenesis.
[54]	Macrophages, dendritic cells, neutrophils, B cells^2^, CD4+ T cells^2^, CD8+ T cells^2^	Mouse	Flow cytometry	ex: 355 nm, em: 425–475 nm (NADH), 540–580 nm (FAD)	Innate immune cells exhibited higher AF compared to adaptive cells, with the highest signals of NADH and FAD observed in dendritic cells.
			Fluorescence imaging, two‐photon excitation	ex: 810 nm, em: 415–485 nm (NADH), em: 540–580 nm (FAD)	
[100]	Granulocytes, leukocytes^3^	Mouse	Fluorescence microscopy, two‐photon excitation	ex: 590 nm, em: 330–380 nm (tryptophan); ex: 730 nm, em: 420–480 nm (NADH)	Granulocytes exhibited tryptophan‐associated AF and low, uneven NADH‐associated signals in granulocytes.
[96]	Mast cells	Mouse	Fluorescence microscopy, two‐photon excitation	ex: 590 nm, em: 330–380 nm (tryptophan)	Tryptophan‐rich granules in mast cells showed sharp AF decrease upon degranulation, with bright AF in vivo indicating a specific dermal subpopulation, lost in mast cell‐deficient mice but restored by bone marrow mast cell injection.
[109]	Neutrophils	Human, mouse	Fluorescence microscopy	ex: 325–375 nm, em: > 419 nm	Neutrophils undergoing phagocytosis exhibited changes in AF heterogeneously distributed across the cell. After stimulation, NAD(P)H‐related AF of neutrophils was reduced.
[95]	Mast cells	Rat	Fluorescence microscopy	ex: 305 nm, em: not reported	Stimulation of mast cells resulted in AF changes due to serotonin release, with a pattern of initial burst followed by sustained release and asynchronous activity within different cell regions.
[58]	Macrophages (J774A)	Cell line	Fluorescence microscopy, two‐photon excitation	ex: 780 nm, em: 400–495 nm, 495–560 nm, 560–680 nm	NAD(P)H AF in macrophages was significantly affected by coverslip coatings and metabolic disruption.
[66]	Macrophages	Rabbit	Time‐resolved laser‐induced fluorescence spectroscopy	ex: 337 nm, em: 360 to 600 nm, 5 nm increments	AF spectral and time‐domain data effectively distinguished macrophage‐rich from collagen‐rich intima in coronary plaques.
[114]	Eosinophils	Human	Spectrofluorimetry	Involves ex: 380 nm, 450 nm, em: 520 nm	Eosinophil AF primarily stemmed from FAD, FMN, and riboflavin, with higher emission compared to neutrophils.
			Fluorescence assay (pH dependence of flavins)	ex: 445 nm, em: 527 nm	
[69]	Macrophages	Zebrafish	FLIM, two‐photon excitation	ex: 750 nm, em: 446–486 nm (NAD(P)H); ex: 895 nm, em: 528–552 nm (FAD)	TNFα+ macrophages had lower NAD(P)H mean lifetimes and a more oxidised state reflected in a lowered optical redox ratio (NAD(P)H/(NAD(P)H + FAD)) in wound healing.
[43]	Monocytes, granulocytes, agranulocytes, neutrophils, eosinophils, lymphocytes^2^	Human	Spectrofluorometry	ex: 250–370 nm (increment: 10 nm), em: 280–600 nm, 200 nm range (shifted 20 nm with respect to ex WL)	Lymphocytes (low intensity), monocytes (intermediate), neutrophils (intermediate), and eosinophils (high) revealed differences in their spectral patterns.
			Microspectro‐fluorometry	ex: 356–376 nm, em: 430–620 nm; ex: 426–446 nm, em: 490–680 nm	
			Fluorescence imaging	ex: 356–376 nm, em: > 430 nm; ex: 426–446 nm, em: > 480 nm	
[112]	Neutrophils	Human	Confocal microscopy	ex: 364 nm, em: 400–550 nm (NAD(P)H)	AF in neutrophils decreased upon phagocytic activation due to NAD(P)H oxidase activity, with various stimuli showing different effects on AF intensity. Neutrophil AF was significantly reduced in bacterial pneumonia compared to healthy controls, with variations across pneumonia types, aiding in distinguishing infection types.
[71]	Macrophages	Human	FLIM, two‐photon excitation	ex: 760 nm, 800 nm; em: 426–490 nm, 502.5–537.5 nm	Macrophage phenotypes were classified with high accuracy, considering donor heterogeneity.
[80]	Alveolar macrophages	Human	FACS	ex: 488 nm, em: > 585 nm	FACS analysis identified brightly autofluorescent alveolar macrophages in smoker lung autopsies, distinguishing them from non‐autofluorescent cells, linking AF to carbon and hemosiderin inclusions and correlating AF with the cells' potential to stimulate leukocytes.
[53]	Macrophages	Human	Flow cytometry	ex: 488 nm, em: 525 nm	AF macrophages developed from monocytes.
[102]	Granulocytes, lymphocytes^2^	Mouse (gut mucosa)	Fluorescence microscopy, two‐photon excitation	ex: 730 nm, em: 380–450 nm, 450–500 nm, 500–580 nm	AF visualised healing in laser‐induced gut mucosal lesions with highly autofluorescent granulocytes.
[78]	Alveolar macrophages	Human	Fluorescence microscopy	ex: 490 nm, em: > 529 nm; ex: 546 nm, em: > 590 nm	High AF in macrophages after PAH exposure indicated sensitivity to cigarette smoke, with nearly all exposed cells showing increased AF.
			Spectral confocal laser scanning microscopy	ex: 490 nm, em: > 529 nm; ex: 546 nm, em: 590 nm	
			FACS	FACS Aria, Becton‐Dickinson (ex: 405, 488, 633 nm; em: 430–470 nm, 515–545 nm, 563–589 nm, 600–620 nm, 675–71 nm, 750–810 nm, 640–680 nm; not all combinations were used)	
[17]	Macrophage (RAW264.7)	Cell line (Mouse)	Quantitative phase microscopy and AF imaging	ex: ~380–396 nm, em: ~414–480 nm	Dynamic AF changes in LPS‐stimulated macrophages were detected and classified on a single‐cell level with stimulation increasing the heterogeneity of cell characteristics.
[79]	Alveolar macrophages	Human	Fluorescence microscopy	ex: 366 nm, em: 390–450 nm	AF intensity in lung macrophages correlated with smoking frequency.
[67]	Macrophages	Human	FLIM	ex: 355 nm, em: 473.5–514.5 nm	AF lifetime correlated with superficial macrophage accumulation in coronary atherosclerotic plaques.
[88]	Microglia	Mouse	Flow cytometry	LSRII cytometer (channels not thoroughly reported, FITC and potentially others)	High AF subpopulation identified in aged brains linked to lipofuscin accumulation and associated with increased hypertrophy, granularity, and oxidative stress with depletion resulting in loss of AF subpopulation which could not be restored upon repopulation.
[93]	Mast cells, neutrophils, eosinophils	Human	Fluorescence microscopy	—	Mast cells, neutrophils, and lymphatic cells showed no AF in IBS biopsies, while eosinophil AF could stage eosinophilia and differentiate Crohn's disease from ulcerative colitis.
[44]	Monocytes (THP‐1), neutrophils (MoT), eosinophils (HL60), T cells^2^ (Jurkat)	Cell lines	Fluorescence microscopy, two‐photon excitation	ex: 700 nm, em: 500–600 nm	Monocytes, neutrophils, and T cells emitted lower AF compared to eosinophils.
[115]	Eosinophils	Human	Fluorescence microscopy	ex: < 546 nm, em: > 580 nm	Fixed bone marrow biopsies of diseased patients showed eosinophil degranulation when analysing their AF.
[118]	Natural killer cells, B cells^2^, T cells^2^	Human	Fluorescence microscopy, two‐photon excitation (uses T cell dataset from [127])	ex: 750 nm, em: 400–480 nm (NAD(P)H); ex: 890 nm, em: 500–600 nm (FAD)	Activated human NK cells showed increased redox ratio, altered NAD(P)H and FAD lifetimes, and were discriminated from quiescent cells with high accuracy as well as from B and T cells, both activated and quiescent.
[75]	Alveolar macrophages	Human	Flow cytometry	—	AF intensity in alveolar macrophages correlated with cell size and granularity, with nonadherent macrophages having higher AF than adherent cells.
[60]	Macrophages	Human	FLIM, two‐photon excitation	ex: 755 nm, em: 440–480 nm (NAD(P)H); ex: 860 nm, em: 500–550 nm (FAD)	Both pro‐inflammatory and anti‐inflammatory stimulation of macrophages increased macrophage redox ratios and altered mitochondrial clustering, with distinct AF signatures used for classification.
[81]	Alveolar macrophages	Cow	Flow cytometry	FACScalibur, Becton Dickinso	AF signatures effectively distinguished alveolar macrophages from neutrophils and lymphocytes, enabling accurate phenotyping without staining.
[76]	Alveolar macrophages	Human	Fluorescence microscopy	ex: 490 nm, em: > 529 nm; ex: 546 nm, em: > 590 nm	AF in lung macrophages varied significantly with smoking status, linked to PAH presence, with smoker macrophages showing higher intensity.
			Flow cytometry	Coulter Corporation, Hialeah, FL, model Profile II	
			Spectrofluorometry	ex: 380 nm, em: ~200–840 nm; ex: ~200–840 nm, em: 430 nm	
[64]	Macrophages	Mouse	FLIM, two‐photon excitation	ex: 780 nm, em: 427.5–462.5 nm (NADH); ex: 890 nm, em: 547–577 nm (FAD)	Tumour‐associated macrophages were identified non‐invasively through their FAD and NADH signatures, highlighting their distinct glycolytic signatures from surrounding tumor cells.
[86]	Alveolar macrophages, neutrophils, eosinophils, lymphocytes^2^	Human	Fluorescence microscopy, two‐photon excitation	ex: 1070 nm, em: 562–665 nm	AF and third harmonic generation data, combined with deep learning, accurately predicted macrophage fractions in BAL fluid. Neutrophils showed less AF than eosinophils with the deep learning model predicting both fractions too low.
			Fluorescence microscopy, three‐photon excitation	ex: 1070 nm, em: 380–420 nm	
[83]	Alveolar macrophages, neutrophils	Hamster	Flow cytometry	ex: 488 nm, em: > 520 nm	High AF in alveolar macrophages confirmed their identity in both control and elastase‐induced emphysema groups, exceeding the intensity measured for bone marrow monocytes and polymorphonuclear neutrophils.
[19]	Neutrophils	Mouse	FACS	FACS II cell sorter, ex: 351.1 nm, 363.8 nm, em: > 425 nm	Neutrophils from peritoneal exudate and bone marrow showed similar emission spectra.
			Spectrofluorimetry	ex: 360 nm, 400–600 nm	
[107]	Neutrophils, eosinophils	Human	Spectrofluorimetry	ex: 450 nm, em: ~490–800 nm; ex: 275–475 nm, em: 520 nm	Eosinophil AF was linked to granules and showed higher signals than neutrophils, aiding in FACS with high purity. AF for healthy and eosinophilia patients differed.
			Fluorescence microscopy	ex: < 500 nm, em: 510–605.5 nm	
			FACS	FACS‐II (Becton Dickenson), ex: 457 nm	
[94]	Mast cells (RBL‐2H3)	Cell line	Fluorescence microscopy, three‐photon excitation	ex: 740 nm, em: 320–400 nm (serotonin)	AF was used to track serotonin release in stimulated mast cells, showing a pattern of fast and slow secretion phases with regional variations within cells.
[46]	Monocytes, granulocytes, neutrophils, eosinophils, erythrophagocytic cells, lymphocytes^2^, leukocytes^3^	Human	Epifluorescence microscopy	ex: 361–389 nm, em: 430–490 nm; ex: 465–495 nm, em: 512.5–557.5 nm; ex: 540–580 nm, em: 600–660 nm	Monocytes emitted weaker AF signals than granulocytes. In blood vessels, erythrocytes absorbed the AF of leukocytes and reduced excitation light penetration. Erythrophagocytic cells displayed red‐shifted AF resulting from the degradation of erythrocytes.
			Confocal microscopy	ex: 405 nm, em: 425–475 nm; ex: 488 nm, em: 500–550 nm; ex: 543 nm, em: 570–620 nm; ex: 638 nm, em: 660–740 nm	
			Flow cytometry	ex: 405 nm, em: 427.5–472.5 nm, em: 505–545 nm; ex: 488 nm, em: 505–545 nm, em: 600–620 nm, em: 665–715 nm	
			FLIM	ex: 402 nm; em: 420–460 nm (NAD(P)H), em: > 520 nm (FAD)	
[65]	Macrophages, immune cells^3^	Mouse	Fluorescence imaging	ex: 375 nm, em: 440–460 nm (NAD(P)H); ex: 488 nm, em: 515–545 nm (FAD)	FAD AF strongly co‐localised with CD markers of M2 macrophages.
[108]	Neutrophils	Zebrafish	Fluorescence spectroscopic imaging, two‐photon excitation	ex: 700 nm, em: 400–600 nm	Neutrophils exhibited a NAD(P)H‐related AF peak at 445 nm upon 700 nm excitation.
[98]	Agranulocytes, granulocytes, neutrophils	Human	Spectroscopic fluorescence imaging, two‐photon excitation	ex: 600 nm, em: ~310–575 nm (Tryptophan); ex: 720 nm, em: ~310–575 nm (NADH)	Neutrophils revealed uniform NADH AF, indicating the absence of mitochondrial networks and reliance on glycolysis.
[99]	Agranulocytes, Granulocytes, neutrophils.	Human	Spectroscopic fluorescence imaging, two‐photon excitation	ex: 600 nm, em: ~310–575 nm (Tryptophan); ex: 720 nm, em: ~310–575 nm (NADH)	AF peaks associated with tryptophan and NADH were detected in granulocytes, and agranulocytes.

**TABLE 2:** Overview on adaptive immunity and their autofluorescent properties (WL: Wavelength, ex: Excitation, em: Emission, AF: Autofluorescence, ^1^innate immune cells, ^3^uncategorised immune cells).

Paper	Cell Type	System	Assessment	WL for AF	Aim/Conclusion
[133]	T cells	Human	FLIM, two‐photon excitation (uses T cell dataset from [127])	ex: 750 nm, em: 400–480 nm (NAD(P)H); ex: 890 nm, em: 500–600 nm (FAD)	Simulated AF data enabled the classification of rare drug‐resistant and anti‐inflammatory T cell populations with high accuracy.
[124]	Lymphocytes	Mouse	Two‐photon fluorescence imaging	ex: 720–750 nm, em: 380–560 nm (NAD(P)H)	Lymphocyte movement in murine Peyer's patches was visualised through AF in response to M cells sampling gut antigens.
[56]	Macrophages^1^, dendritic cells^1^, B cells, T cells	Mouse	FLIM, two‐photon excitation	ex in vitro: 710 to 830 nm, ex in vivo: 710 to 750 nm, em: 350 to 600 nm	B cells showed distinct AF intensities and lifetimes compared to T cells, dendrites, and macrophages. AF of both B and T cells changed upon activation.
			FLIM, two‐photon excitation	ex: 710, 730, 750, 780, 800 and 830 nm, em: 380–450 nm, 450–500 nm, 500–580 nm, 580–680 nm	
[129]	T cells	Human	Confocal microscopy	ex: 480 nm, em: > 530 nm	Lipofuscin accumulation in long‐term cultured T cells led to increased AF, correlating with autophagy and cell death.
[121]	Lymphocytes	Human	Fluorescence microscopy	ex: < 500 nm, em: > 515 nm	Lymphocytes from patients with neurodegenerative diseases displayed AF due to cytoplasmic inclusions, associated with specific conditions like neuronal ceroid lipofuscinosis.
[128]	T cells	Human	FLIM, two‐photon excitation (uses T cell dataset from [127])	ex: 750 nm, em: 400–480 nm (NAD(P)H); ex: 890 nm, em: 500–600 nm (FAD)	Activated T cells showed lower cytoplasmic redox ratios compared to mitochondrial regions, with limited correlation between intensity and lifetime‐based measurements.
[130]	T cell	Mouse	FLIM, two‐photon excitation	ex: 750 nm, em: 450–490 nm (NAD(P)H); ex: 900 nm, em: 500–550 nm (FAD)	T cells in large tumour‐bearing mice had higher mean NAD(P)H fluorescence lifetimes, linked to NADPH biosynthesis.
			FLIM, single‐photon excitation	ex: 488 nm, em: 500–570 nm (EGFP)	
[131]	T cell	Mouse	FLIM, two‐photon and single‐photon excitation	ex: 750 nm, em: 450–490 nm (NAD(P)H)	Responders to immunotherapy had higher amplitude ratio of free NADH to protein‐bound NAD(P)H, potentially indicating metabolic shifts.
			FLIM, single‐photon excitation	ex: 488 nm, em: 500–570 nm (EGFP)	
[85]	Alveolar macrophages^1^, lymphocytes, leukocytes^3^	Mouse, human	Flow cytometry	CytoFLEX LX, Cytek Aurora 5 L	Fixed blood lymphocytes exhibited higher AF levels than live ones for UV, violet, and blue excitation in spectral flow cytometry
[103]	Granulocytes^1^, lymphocytes	Mouse	Fluorescence microscopy, two‐photon excitation	ex: (1) 800 nm (band width > 150 nm), (2) 730–800 nm (tunable), em: 396–452 nm, 446–499 nm, 499–582 nm, 583–671 nm	Lymphocytes were identified by their dark nuclei and thin autofluorescent cytoplasm, facilitating their recognition combined with size and speed.
[54]	Macrophages^1^, dendritic cells^1^, neutrophils^1^, B cells, T cells (CD4+, CD8+)	Mouse	Flow cytometry	ex: 355 nm; em: 425–475 nm (NADH), em: 540–580 nm (FAD)	B and T cells had lower NADH and FAD fluorescence than dendritic cells, with increased NADH and FAD upon stimulation, significantly impacting the redox ratio. T cell AF differed between CD8+ and CD4+ cells.
			Fluorescence imaging, two‐photon excitation	ex: 810 nm, em: 415–485 nm (NADH), em: 540–580 nm (FAD)	
[43]	Monocytes^1^, granulocytes^1^, agranulocytes^1^, neutrophils^1^, eosinophils^1^, lymphocytes	Human	Spectrofluorometry	ex: 250–370 nm (increment: 10 nm), em: 280–600 nm, 200 nm range (shifted 20 nm w/respect to ex WL)	Lymphocytes exhibited the lowest AF intensity among leukocytes (eosinophils, neutrophils, monocytes), with homogeneous cytoplasmic distribution of AF
			Microspectrofluorometry	ex: 356–376 nm, em: 430–620 nm; ex: 426–446 nm, em: 490–680 nm	
			Fluorescence imaging	ex: 356–376 nm, em: > 430 nm; ex: 426–446 nm, em: > 480 nm	
[102]	Granulocytes^1^, lymphocytes	Mouse (gut mucosa)	Fluorescence microscopy, two‐photon excitation	ex: 730 nm, em: 380–450 nm, 450–500 nm, 500–580 nm	Lymphocytes appeared dark due to non‐AF nuclei, aiding in identification in heat‐damaged murine gut mucosa tissue.
[120]	Lymphocytes	Human	Flow cytometry	ex: 488 nm, em: 515–545 nm, 564–606 nm, 670–735 nm, 750–810 nm; ex: 633/640 nm, em: 650–670 nm, 750–810 nm; ex: 405 nm, em: 425–475 nm, 485–535 nm	Lymphocytes exposed to medicinal plant extracts showed increased AF and were positive for activation markers.
[126]	B cells (B‐lymphoma cell line CA46), T cells	Cell line, Human	Confocal microscopy	Em scanning, 5 nm increments: ex: 351 nm, em: 400–600 nm; ex: 458 nm, em: 512–600 nm; ex: 488 nm, em: 512–600 nm Total em range: ex: 488 nm, em: 520–580 nm	In vitro cultured B lymphoma cell line had distinct AF spectra, with higher fluorescence compared to T cells under certain excitations (488 nm), useful for in vivo localisation.
[123]	Lymphocytes	Human	Fluorescence microscopy	ex 365 nm; em: 425–475 nm, 525–575 nm, 629–679 nm	Human lymphocytes from excised lymph node tissue had weak AF signals due to large non‐AF nuclei, with no differences in AF observed between lymphocytes from lymphoid follicles and lymphoma cells.
[44]	Monocytes^1^ (THP‐1), neutrophils^1^ (MoT), eosinophils^1^ (HL60), T cells (Jurkat)	Cell lines	Fluorescence microscopy, two‐photon excitation	ex: 700 nm, em: 500–600 nm	Immortalised T cells had less AF than eosinophils.
[135]	T cells	Human	FLIM	ex: 375 nm, em: 415–485 nm (NAD(P)H)	High classification accuracy was achieved for T cells measured on a cost‐efficient device.
[118]	Natural killer cells^1^, B cells, T cells	Human	Fluorescence microscopy, two‐photon excitation (uses T cell dataset from [127])	ex: 750 nm, em: 400–480 nm (NAD(P)H); ex: 890 nm, em: 500–600 nm (FAD)	Activated B cells exhibited a higher redox ratio and were discriminated from quiescent B cells and other immune cells (NK, T cell) with high accuracy considering both cell type and activation status.
[122]	Lymphocytes	Mouse	Two‐photon fluorescence imaging	ex: 730 nm, em: not provided	In ocular CALT, stimulated lymphocytes exhibited dark nuclei with a thin autofluorescent rim, aiding in visualisation within lymphoid follicles.
[86]	Alveolar macrophages^1^, neutrophils^1^, eosinophiles^1^, lymphocytes	Human	Fluorescence microscopy, two‐photon excitation	ex: 1070 nm, em: 562–665 nm	A deep learning model using AF data identified fractions of lymphocytes, eosinophils, neutrophils, and macrophages.
			Fluorescence microscopy, three‐photon excitation	ex: 1070 nm, em: 380–420 nm	
[127]	T cells	Human	FLIM, two‐photon excitation	ex: 750 nm, em: 400–480 nm (NAD(P)H); ex: 890 nm, em: 500–600 nm (FAD)	Significant AF changes, including increased NAD(P)H and changes in redox ratios, were observed and described in detail for T cell activation. Changes were consistent across multiple donors and resulted in highly accurate classification.
[134]	T cells	Human	FLIM, two‐photon excitation (uses T cell dataset from [127])	ex: 750 nm, em: 400–480 nm (NAD(P)H); ex: 890 nm, em: 500–600 nm (FAD)	Deep learning models achieved high accuracy in classifying quiescent and activated T cells from AF data.
[46]	Monocytes^1^, granulocytes^1^, neutrophils^1^, eosinophils^1^, erythrophagocytic cells^1^, lymphocytes, leukocytes^3^	Human	Flow cytometry	ex: 405 nm, em: 427.5–472.5 nm, em: 505–545 nm; ex: 488 nm, em: 505–545 nm, em: 600–620 nm, em: 665–715 nm	Lymphocytes could be distinguished from monocytes and granulocytes.

**TABLE 3:** Overview on uncategorised immune cells and their autofluorescent properties (WL: Wavelength, ex: Excitation, em: Emission, AF: Autofluorescence, ^1^innate immune cells, ^2^adaptive immune cells).

Paper	Cell type	System	Assessment	WL for AF	Aim/conclusion
[136]	Mixture of monocytes^1^, polarised macrophages^1^ (RAW264.7), neutrophils^1^	Human, cell line	Metabolic oscillations (NAD(P)H)	ex: 355–375 nm, em: 393–417 nm (NAD(P)H)	AF amplitude and frequency changed with different stimulants.
[82]	Alveolar macrophages^1^, neutrophils^1^, leukocytes	Mouse (lymph nodes, BAL, lung tissue)	Flow cytometry	Cytek Aurora 5	BAL leukocytes had higher AF than leukocytes from lung tissue and lymph nodes with AF profiles changed with regards to infection type and inflammation.
[139]	Immune cells	Mouse	FLIM, two‐photon excitation	ex: 750 nm, em: 426–464 nm (NAD(P)H); ex: 750 nm + 1041 nm = 872 nm (two‐color two‐photon ex), em: 499–529 nm (FAD)	Tumor‐infiltrating immune cells were larger with reduced FAD and NAD(P)H lifetimes compared to tumour cells.
[85]	Alveolar macrophages^1^, lymphocytes^2^, leukocytes	Mouse, human	Flow cytometry	CytoFLEX LX, Cytek Aurora 5 L	Chronic and acute viral infections showed distinct leukocyte AF profiles despite similar spectra.
[138]	Immune cells	Mouse	Fluorescence microscopy, two‐photon excitation	ex: 810 nm, em: 415–485 nm (NADH), 540–580 (FAD)	In murine pulmonary fibrosis, AF changes in NADH (decrease) and FAD (increase) correlated with disease severity.
[54]	Macrophages^1^, dendritic cells^1^, neutrophils^1^, B cells^2^, CD4+ T cells^2^, CD8+ T cells^2^	Mouse	Flow cytometry	ex: 355 nm, em: 425–475 nm (NADH), 540–580 nm (FAD)	Innate immune cells showed higher AF than adaptive cells at 355 nm excitation. Cell death resulted in a lowered NADH AF.
			Fluorescence microscopy, two‐photon excitation	ex: 810 nm, em: 415–485 nm (NADH), 540–580 nm (FAD)	
[100]	Granulocytes^1^, leukocytes	Mouse	Fluorescence microscopy, two‐photon excitation	ex: 590 nm, em: 330–380 nm (tryptophan); ex: 730 nm, em: 420–480 nm (NADH)	Tryptophan‐associated AF and low NADH signal in granulocytes aided in tracking leukocyte movement and inflammation visualisation.
[137]	Immune cells	Mouse	Fluorescence imaging	ex: 800 nm, em: 450–500 nm (collagen), em: 500–580 nm	Immune cell migration toward inflammation sites was tracked with regards to behaviour and velocity using cytoplasmic AF.
[46]	Monocytes^1^, granulocytes^1^, neutrophils^1^, eosinophils^1^, erythrophagocytic cells^1^, lymphocytes^2^, leukocytes	Human	Epifluorescence microscopy	ex: 361–389 nm, em: 430–490 nm; ex: 465–495 nm, em: 512.5–557.5 nm; ex: 540–580 nm, em: 600–660 nm	AF differentiated erythrophagocytic cells and leukocytes, with FAD channel signals observed in FLIM but no NAD(P)H channel detection.
			Confocal microscopy	ex: 405 nm, em: 425–475 nm; ex: 488 nm, em: 500–550 nm; ex: 543 nm, em: 570–620 nm; ex: 638 nm, em: 660–740 nm	
			Flow cytometry	ex: 405 nm, em: 427.5–472.5 nm, 505–545 nm; ex: 488 nm, em: 505–545 nm, 600–620 nm, 665–715 nm	
			FLIM	ex: 402 nm, em: 420–460 nm (NAD(P)H), > 520 nm (FAD)	
[65]	Macrophages^1^, immune cells	Mouse	Fluorescence imaging	ex: 375 nm, em: 440–460 nm (NAD(P)H); ex: 488 nm, em: 515–545 nm (FAD)	FAD AF highlighted immunosuppressive cells, while NAD(P)H AF identified cancer and immunoactive cells, aiding in distinguishing immune responses during immunotherapy.

The following section presents immune cell autofluorescence results, categorised by cell type. The information is structured to address cell characterisation based on autofluorescent signals, the effect of pathological states on cellular autofluorescence, and the identification of cell type and activation status through immune cell autofluorescence.

### 3.1 Innate Immunity

Innate immunity serves as the body's first line of defence, providing a rapid and non‐specific response to pathogens through physical barriers, immune cells, and soluble factors that help to contain infections as a first defence and subsequently help shape adaptive immunity [41]. Immune cell types which contribute to the innate immune system and have had their autofluorescence studied, include monocytes, macrophages, and some tissue resident macrophages including alveolar macrophages and microglia, dendritic cells, mast cells, granulocytes neutrophils, eosinophils, erythrophagocytic cells, and natural killer cells (Table 1).

#### 3.1.1 Monocytes

Monocytes, produced in the bone marrow circulate in the bloodstream, and can differentiate into macrophages, aiding in pathogen clearance and tissue repair with a typical lifespan of 1–2 days without activation [42].

##### 3.1.1.1 Characterisation

Autofluorescence characteristics of monocytes were explored by Monici et al. through spectrofluorometry and fluorescence image analysis. Autofluorescent signal helped in distinguishing cell types by both intensity and spectral shape of the cell. Monocytes and neutrophils emitted similar intermediate fluorescence intensity levels when compared to lymphocytes (low intensity) and eosinophils (high intensity) with signatures speculated to involve the native fluorophores NADH (free and bound) and flavins [43]. In a cell line experiment, the signal emitted by monocytes (THP‐1) was lower than neutrophils (MoT) and T cells (Jurkat) and significantly lower than eosinophils (HL60) in two‐photon fluorescence imaging [44]. Monocytes showed distinct autofluorescence patterns compared to neutrophils, with monocytes displaying higher autofluorescence at 366 nm excitation and neutrophils at 436 nm [43]. In this study, cells were isolated based on their plastic adherence properties, which may not represent the entire monocyte population and affect spectral properties. This is because monocytes exist as a mixture of adherent and non‐adherent cells [45], with adherence often indicating differentiation towards macrophages. In flow cytometry, the average intensity emitted by human monocytes was half the signal of granulocytes for various excitation/emission ranges, with a slight increase in signal upon 488 nm excitation and 505–545 nm emission [46]. Monocyte activation resulted in stronger and spectrally altered autofluorescent signals, as demonstrated in Adachi et al.'s experiments [47] by stimulation using IFN‐γ, further enhanced through the addition of human CpG DNA.

##### 3.1.1.2 Pathological States

In 1995, Glenn et al. [48] investigated native autofluorescence low‐density lipoproteins (LDL) and reported distinctive spectral differences at 308 nm excitation between oxidatively modified LDL and its native non‐oxidised form. When applying their findings to human monocytes from individuals with normal versus elevated cholesterol levels, significantly higher autofluorescence was measured in the latter suggesting an accumulation of oxidised LDL associated with atherosclerosis [48, 49]. Monocytes co‐cultured with breast cancer cells showed autofluorescent signatures consistent with macrophages indicating cell differentiation in the presence of tumours as reported in [50].

#### 3.1.2 Macrophages

Macrophages, derived from monocytes, are essential in the detection, phagocytosis, and destruction of pathogens [51, 52]. They also play a key role in triggering inflammation processes and initiate tissue repair [51]. Subtypes of macrophages include unpolarised M0 macrophages, pro‐inflammatory M1 macrophages and anti‐inflammatory M2 macrophages [51].

##### 3.1.2.1 Characterisation

Monitoring the autofluorescence of human PBMCs maintained in vitro over time using flow cytometry, Njoroge et al. [53] observed an autofluorescent subpopulation of relatively large cells with high side scatter, indicative of increased cellular complexity or granularity. The study examined the effect of depleting adherent cells at the start of the experiment. In the non‐depleted group, the autofluorescent subpopulation expanded from 0.5% on day 1%–14% on day 5 under unstimulated conditions, before receding to 6% on day 8. The initial depletion of adherent monocytes on day 1 significantly impeded the development of this subpopulation. Staining (Giemsa and immune markers HLA‐DR, lysozyme, CD68, CD20 and CD4) confirmed macrophage identity and that the cells were capable of phagocytosing 10 μm plastic beads. To understand the transcriptomic profile of this cell, 12 cytokines were assessed, with only IL‐1α significantly upregulated, indicating macrophage population, compared to other PBMCs. High autofluorescence in macrophages compared to other immune cells—including increase on stimulation—was mirrored by Lemire 2022 [54]. Murine immune cell types (CD4+ T cells, CD8+ T cells, B cells, macrophages, dendritic cells, neutrophils), were assessed using flow cytometry. Macrophages were found to have significantly elevated NAD(P)H compared to CD8+ T cells, and significantly elevated FAD compared to both CD8+ and CD4+ T cells. Polarisation of macrophages significantly increased NAD(P)H (2.9x), however the type of polarisation (M0, M1, M2) did not affect this. When cells were reinvestigated using multiphoton microscopy, results were reproduced, with an increase of NAD(P)H and FAD upon stimulation being particularly apparent in macrophages. In another study using flow cytometry, Lei et al. also examined immortalised mouse macrophage dynamics [55] and found macrophages behaved similar to microglia (section: Microglia) in the phagocytosis of rod outer segments (i.e., macrophages fed with rod outer segments and rod outer segment components had increased lipofuscin‐like autofluorescence which was highest for oxidised rod outer segments).

In vitro cultured murine macrophages were shown to have higher autofluorescence than any other cell types (T cells, B cells, dendritic cells) using two‐photon FLIM at all excitation wavelengths assessed [56]. In addition, autofluorescence lifetimes of macrophages differed significantly from other cell types depending on the excitation wavelength: at 710 nm compared to dendritic cells and lymphocytes, at 750 nm compared to dendritic cells and B cells, and at 800 nm compared to T cells. Two‐photon microscopy used for the detection of NAD(P)H in RAW264.7 macrophages [57] further demonstrated that exposure to glucose or NaCN (an inhibitor of cellular respiration) evoked an increase in apparent NAD(P)H autofluorescence, while activators of NADPH oxidase (TNF‐α, H_2_O_2_, phorbol myristate acetate (PMA), NaF) resulted in a significant decrease. Coverslip coating (normal, fibronectin or Poly‐D‐lysine) [58] significantly affected signals in the two‐photon NAD(P)H autofluorescence microscopy (excitation 780 nm, emission 400–495 nm, 495–560 nm, 560–680 nm) of in vitro cultured J774A macrophages. The disruption of normal metabolism by exposure to saturated potassium solution could also be detected by this system [58]. The experiments could have been strengthened by adjusting the excitation and emission wavelengths, as the selected wavelengths did not ideally align with the properties of the investigated NAD(P)H. The oscillation period of NAD(P)H in RAW264.7 macrophages and human monocytes was measured to be approximately 20 s [47]. Activation by IFN‐γ and CpG DNA resulted in increased oscillation amplitude as well as frequency. A phase matched electric field could also increase NAD(P)H oscillatory amplitude.

Bourdely et al. observed subpopulations of macrophages with high and low autofluorescence in murine and human systems [59]. IL‐6 was higher in autofluorescent macrophages in both stimulated and unstimulated conditions. A higher proportion of autofluorescent macrophages were TNF‐α positive after stimulation (TLR4 engagement) compared to non‐autofluorescent macrophages (36.5% vs. 20.5%)—although under basal conditions both populations were low TNF producers [59]. The exposure of human macrophages to pro‐inflammatory stimulation via LPS or anti‐inflammatory stimulation via IL‐4 (studied using two‐photon lifetime imaging microscopy targeting NAD(P)H and FAD) resulted in significantly increased redox ratios (FAD/[FAD+NAD(P)H]) for both pro‐ and anti‐inflammatory treatments, with the increase being highest for the pro‐inflammatory conditions [60]. Mitochondrial clustering was also decreased for both treatments. There were significant differences when macrophages were exposed to the stimuli for 6 or 24 h (greater change for longer exposure), though unstimulated macrophages were not affected by time, and NAD(P)H bound fraction did not change with exposure. However, when Alfonso‐Garcia et al. investigated the effect of polarisation (stimulated by IL‐4 with IL‐13) on the metabolism of mouse bone marrow derived macrophages using two‐photon FLIM [61], found a higher contribution to their autofluorescence from bound NADH—indicative of increased glycolytic metabolism. When RAW264.7 mouse macrophages were stimulated with IFN‐γ (stimulating an anti‐tumour M1‐like phenotype), IL‐4/IL‐13 (stimulating a pro‐tumour M2‐like phenotype), or left unstimulated (naïve) [50], NAD(P)H and FAD autofluorescence showed a time dependent difference between the IFN‐γ and the IL‐4/IL‐13 macrophages. The redox ratio was significantly different between the three stimulation states at 48 and 72 h. NAD(P)H and FAD were also dynamic across the conditions, and consistently exhibited significant differences 24 h post‐stimulation. In Gehlsen et al., when macrophages were activated by PMA exposure relative intensity of autofluorescence at 750 nm was significantly decreased, while lifetime was increased [56].

##### 3.1.2.2 Pathological States

Tumour‐associated macrophages are a part of the tumour microenvironment (TME) and exert a significant influence on tumour growth and metastasis [62]. One study using spectral flow cytometry to study mouse and human macrophages from perilesional skin and cutaneous squamous cell carcinoma found that autofluorescent cells represented a highly phagocytic subtype compared to their non‐autofluorescent counterpart [59]. Autofluorescent macrophages were also shown to express high levels of the CD206 mannose receptor—a phenotypic marker for phagocytic macrophages in intestinal tissue and bone marrow. Additionally, they had high endocytic capacity relative to non‐autofluorescent macrophages. Cell markers indicated that autofluorescent macrophages originated from prenatal haematopoiesis or adult monocytes in skin. Two‐photon in vivo microscopy targeting NAD(P)H, FAD and the mCherry region (567.5–612.5 nm, avoiding spectral overlap with endogenous fluorophores) showed that tumour associated mouse macrophages were more oxidised than dermal ones. The mCherry fluorescence from a reporter gene was used to confirm macrophage identity [63]. Changes of NAD(P)H and FAD towards longer mean fluorescence lifetimes in dermal macrophages suggest that this reflects differences in NAD(P)H and FAD protein bindings. Dermal macrophages had greater heterogeneity in their optical redox ratio, NAD(P)H and FAD mean lifetimes than tumour macrophages. This finding was replicated using markers of macrophage phenotype (CD206 and CD86). Bourdely et al. observed both high and low autofluorescence macrophages in human peri‐tumoral skin and cutaneous squamous cell carcinoma [59], where autofluorescence identified macrophage subsets expressing the CD206 mannose receptor. In mice, all autofluorescent macrophages expressed CD206, and a subset of those also expressed TIM‐4, a marker of homeostasis in macrophages.

In a 3D microfluidic model recapitulating the breast cancer TME, macrophages (RAW264.7 mouse cell line) and monocytes (THP‐1 human cell line) were cultured alone and with breast cancer cells of the respective species [50]. Two‐photon autofluorescence imaging of NAD(P)H and FAD was used to assess metabolic changes. In mouse cell experiments, co‐culturing RAW264.7 macrophages with primary Polyoma‐Middle T Virus mammary carcinoma cells in the model for 24, 48 and 72 h resulted in altered FAD mean lifetimes and increased immune cell migration towards the tumour layer. Similar effects were observed in human cell co‐cultures, when THP‐1 human monocyte cell line were cultured with patient‐derived invasive breast carcinoma cells. Monocyte derived macrophages had higher NAD(P)H/FAD redox ratios than passively migrating monocytes at 24 and 48 h, demonstrating metabolic heterogeneity between subpopulations—although this effect was lost at 72 h. NAD(P)H mean fluorescence lifetime remained unchanged at all time points, while FAD mean fluorescence lifetime was consistently significantly lower in the tumour model compared to monoculture. The co‐culture with tumour cells used in the 3D culture models (mouse cells, patient derived breast cancer, and triple‐negative breast cancer cells) resulted in metabolic heterogeneity detected in macrophages over the time course, highlighting cancer origin as a source of variability. Szulczewski et al. applied FLIM for the assessment of NADH and FAD to non‐invasively identify tumour associated macrophages in the intact murine mammary TME [64]. A high portion of macrophages had elevated FAD. However, not all cells with high FAD were positive for the resident tissue macrophage marker F4/F80, potentially indicating the presence of an additional subpopulation of high FAD cells in the mammary TME. Overall, macrophages had a more glycolytic NADH‐FLIM signature which made them distinct from surrounding tumour cells. As macrophage recruitment is a significant prognostic factor, the authors concluded that FLIM assessment could have clinical applications. Another study [65] explored the use of autofluorescence to assess the immune response to immunotherapy in a mouse model of triple‐negative breast cancer. The study observed a strong co‐localisation between cells with high emission in the FAD channel and CD206‐PE, CD11b, and CD301b positivity marking M2 macrophages. Macrophages contribute to the formation of plaques and progression of coronary heart disease, which was investigated using time‐resolved laser induced fluorescence spectroscopy in an in vivo rabbit atherosclerosis model [66]. Spectral and time‐domain emission data of plaques distinguished intima rich in macrophage foam cells from intima rich in collagen with high sensitivity (> 85%) and specificity (> 95%). In an investigation of coronary intima autofluorescence lifetime (in fresh postmortem coronary segments), it was found plaque autofluorescence lifetime was correlated with superficial macrophage accumulation in coronary atherosclerotic plaques [67]. Comparing AF signals to gold standard CD68 immunostaining resulted in over 85% accuracy, sensitivity and specificity for classifying plaque when thresholding autofluorescence lifetime at 6 ns. In Glenn et al.'s laser induced fluorescence spectroscopy approach, macrophages (from P388D1) incubated with oxidised low density lipoprotein (LDL) were found to take on its spectral characteristics [48]. The authors concluded that this could be useful for the assessment of atherosclerosis which is associated with focal accumulations of LDL.

In an investigation of prediabetes and diabetes on the autofluorescence properties of mouse adipose tissue, two‐photon FLIM targeting NAD(P)H, FAD, and lipofuscin it was found that lipofuscin‐like autofluorescence was specific to macrophages and distinguished them from adipocytes, suggesting a potential for label‐free assessment of inflammation processes in adipose tissue [68]. Macrophages' redox ratio (FAD/[NAD(P)H + FAD]) and proportion of free NAD(P)H was not affected by the presence of pre‐diabetes in contrast to significant alterations observed in adipocytes. However, in diabetic mice the macrophage redox ratio was significantly reduced, and free NAD(P)H was significantly increased. Lipofuscin‐like autofluorescence was also significantly increased in the macrophages of diabetic mice. Modelling of this data was able to discriminate non‐diabetic mice from mice on a high fat or high fat high sucrose diet with accuracies of 0.86 and 0.88, respectively [68].

Macrophage dynamics were also studied in a zebrafish wound healing model. Two‐photon intensity and lifetime imaging targeting NAD(P)H and FAD showed that TNFα positive macrophages (proinflammatory) had lower NAD(P)H mean lifetime and were more oxidised compared to TNFα negative [69]. Lower optical redox ratio and OMI index (optical metabolic imaging index, see [70] for definition), was observed, with lower NAD(P)H mean lifetime, short lifetime and long lifetime. The authors noted that this may reflect a reduction in glycolytic activity, as these changes were similar to what they found with 2‐deoxy‐d‐glucose (2‐DG) inhibition of glycolysis [69]. Infection and thermal injury induced the development of a macrophage population with a more oxidised redox state in wounded tissues. NAD(P)H mean, short, and long fluorescence lifetime were significantly reduced in macrophages at the Listeris monocytogenes (Lm) infected wound; however, there were no changes in FAD. In contrast, for mice, in vitro infection of macrophages by Lm resulted in increased optical redox ratio. The optical redox ratio and OMI index of macrophages increased over time at both wound types resulting in a more reduced redox state. Treatment with metformin reduced intracellular redox state and the population of TNFα+ wound macrophages resulting in improved tissue repair [69]. Assessment of the living conjunctiva (BALB/c mice) in Gehlsen et al. showed that macrophages had higher integral autofluorescence intensity in inflamed tissue compared to non‐inflamed tissue at 710 and 750 nm [56]. In vivo autofluorescence intensity values for macrophages were lower than in vitro macrophages [56].

In flow cytometry, murine macrophages had the smallest decrease in NAD(P)H upon cell death compared to other immune cell types (CD4+ and CD8+ T cells, B cells, dendritic cells, neutrophils) investigated while FAD was not affected by cell death across all cell types [54].

##### 3.1.2.3 Identification

Two‐photon fluorescence lifetime imaging microscopy (NAD(P)H and FAD^+^ bands) was used to assess circulating monocytes in humans, to identify polarisation into M1 and M2 macrophages through IFN‐γ or IL‐4 stimulation [71]. Uniform Manifold Approximate and Projection machine learning (UMAP), random forests, SVM and logistic regression were used to construct a model which discriminated between the two cell fates, achieving an ROC AUC value of 0.944 and an out‐of‐bag (OOB) error rate of 16.67%. M1 and M2 macrophages from six different donors were investigated to determine the impact of heterogeneity [71]. From these, four donors had acceptable predicting performance, defined as OOB < 31% and ROC AUC > 0.75. Another study investigated the use of macrophage autofluorescence for the classification of six polarisation states on the mouse macrophage cell line RAW264.7 [72] using multi‐channel flow cytometry. Supervised machine learning was able to discriminate all 6 macrophage phenotypes (M0, M1, M2a, M2b, M2c and M2d) with 75.8% accuracy. When the number of phenotypes being identified was restricted to two, three, four, and five, average accuracy was 92.0%, 91.9%, 84.2%, and 80.4%, respectively [72]. Low concentration DAPI staining (0.5 μg/mL) was used to exclude dead cells without interfering with the collection of autofluorescence data as it is not taken up by living cells. M2b macrophages had the most unique autofluorescence signatures—likely due to their granularity. Further, there was lowest confusion between M0, M2b, M1 and M2d macrophages, and highest between M0, M2c and M2a. Difficulty in discriminating between M0, M2c and M2a was encountered by the study, consistent with the high similarity in their gene and protein expression profiles [72].

Using morphological variables acquired from quantitative phase microscopy and autofluorescence images, Pavillon et al. investigated dynamic changes in Raw264 macrophages in response to LPS stimulation to detect activation at a single cell level [17]. In the untreated control cells, 96.38% were identified as such, while in stimulated cells 69.87% were correctly identified. A higher dose of LPS (10, 50, 1000 ng/mL) increased the apparent stimulation as assessed by the model, while co‐treatment with a stimulation inhibitor (progesterone) decreased it. Stimulated cells were noted to have a higher spread of characteristics than unstimulated, suggesting a high degree of cell responses within the activation state. In Reference [60], canonical linear discriminant analysis was used to model the metabolic data classifying macrophages at different exposure times (control, anti‐inflammatory, pro‐inflammatory) at 6 h (71.1% accuracy) and 24 h (83.3% accuracy). In [50], a model derived using multiclass random forest classifiers was able to differentiate passive from actively migrating macrophages with ≥ 85% accuracy in co‐culture with mouse breast cancer cells and > 83% accuracy when co‐cultured with primary human tumour cells. Furthermore, the discrimination of naïve, IFN‐γ stimulated and IL‐4/IL‐13 stimulated macrophages at three timepoints post exposure achieved > 84%.

#### 3.1.3 Alveolar Macrophages

Alveolar macrophages reside in the pulmonary alveoli and are the first line of defence against airborne pathogens. They clear debris, maintain lung homeostasis, and regulate immune responses in the respiratory system [73].

##### 3.1.3.1 Characterisation

In fixed human paediatric bronchoalveolar lavage (BAL) samples, alveolar macrophages showed variable and weak autofluorescence in laser scanning cytometry, preventing the detection of individual alveolar macrophages [74]. Instead, cell clumps were identified, with their autofluorescence showing no correlation with the donor's age [74]. Sköld et al. found that autofluorescence intensity in alveolar macrophages from BAL correlated with increasing cell size and complexity, with the largest and most granular cells exhibiting the strongest fluorescence, though specific excitation/emission wavelengths were not reported for their flow cytometry set‐up [75]. Notably, nonadherent alveolar macrophages displayed higher autofluorescence across all regions compared to the total population, likely due to a greater number of cytoplasmic inclusions [75]. Furthermore, these nonadherent alveolar macrophages showed reduced phagocytic capacity compared to their adherent counterparts [75].

##### 3.1.3.2 Pathological States

###### 3.1.3.2.1 Cancer

High autofluorescence was measured for resident lung macrophages from both healthy and tumour tissue received from living and post‐mortem donors, with result not visibly affected by photobleaching or long‐term culture [76]. Spectrofluorometric analysis revealed a peak at 388 nm for an emission scan at 380 nm excitation, and maximum excitation signal at 375.5 nm when emission was set to 430 nm, when these lung macrophages were disrupted in a chloroform‐methanol solution [76].

###### 3.1.3.2.2 Smoking

Alveolar macrophages in BAL of daily smokers showed red‐brown cytoplasm autofluorescence in epifluorescence microscopy independent from intracellular particle deposits such as coal and silica dust [77]. Lung tissue biopsies of these patients also displayed this autofluorescence, which varied with location and patient age [77]. Similarly, Streck et al. observed that over 95% of lung macrophages from live residual tissue of primary lung tumour patients displayed strong autofluorescence in epifluorescence microscopy [76]. Macrophages from non‐smokers appeared egg‐yolk in colour, while those from habitual smokers had a golden shine. Flow cytometry confirmed high autofluorescence of these alveolar macrophages, particularly from smoker samples, compared to minimal or no signal from hematopoietic cells lines, e.g., T cells and B cells and blood monocytes [76]. Fluorescent signals were also detected for excitation 546 nm and emission > 590 nm [76]. Pauly et al. also showed that macrophages in non‐neoplastic tissue samples from lung tumour patients (smokers), as well as their corresponding dried touch imprints, showed strong autofluorescence under fluorescence microscopy [78]. While the alveolar macrophage autofluorescence varied in multispectral cytometry, blood leukocytes only showed minor autofluorescence signals [78]. The increased signal from smoker macrophages was attributed to the presence of polycyclic aromatic hydrocarbons (PAH), such as tobacco tar, within the cytoplasm [76]. After a 30‐min exposure of isolated macrophages to serum containing PAHs from cigarette smoke, 99.4% of the cells exhibited autofluorescence in fluorescence‐activated cell sorting (FACS) analysis, compared to just 2.3% in the control medium [78]. In confocal laser scanning microscopy, ingested carbon particles inside living macrophages appeared dark against the highly autofluorescent cytoplasm [76]. Fluorescence microscopy of sudanophilic substances extracted from lung macrophages during autopsy using Folch's solution identified smokers through milky green‐orange autofluorescence when excited at 366 nm [79]. While the emission colour remained constant, signal intensity correlated with smoking frequency with no signal emitted for non‐smokers [79]. In loosely adherent mononuclear cells of human smoker lungs with primary carcinoma collected postmortem, FACS identified a brightly autofluorescent population, mainly consisting of alveolar macrophages (nonspecific esterase positive, MO_2_ negative) [80]. These cells contained inclusions of carbon particles and hemosiderin‐laden pigments, distinguishing them from non‐autofluorescent cells [80]. Cell sorting facilitated grouping the alveolar macrophages into autofluorescent poor accessory cells and non‐autofluorescent cells with high potential of stimulating leukocytes [80].

###### 3.1.3.2.3 Respiratory Disease

In flow cytometry, Soethout et al. effectively distinguished alveolar macrophages from BAL of calves with respiratory disease and healthy controls from stained neutrophils and lymphocytes based on their autofluorescence signatures [81]. This enabled accurate phenotyping without the use of macrophage stains that could non‐specifically bind to other cell populations [81]. For stained alveolar macrophages from BAL of pneumonia‐infected mice, accounting for their high AF signal prevented errors in unmixing strategies, thereby ensuring accurate quantification of alveolar macrophages [82]. In a study using flow cytometry, Verghese et al. analysed BAL from hamsters treated with saline and pancreatic elastase [83]. In the saline‐treated control group, over 99% of cells were highly autofluorescent alveolar macrophages, exceeding the autofluorescence observed in bone marrow monocytes. In the elastase‐induced emphysema group, 80% of cells displayed similar autofluorescence and scattering properties, confirming their identity as alveolar macrophages. The remaining cells, primarily polymorphonuclear neutrophils, demonstrated lower scatter and 4–5 times less autofluorescence intensity [83]. Using flow cytometry, Berndt and Müller identified macrophages in non‐lymphocytic cells of porcine BAL by their high green autofluorescence, contrasting with the low autofluorescence associated with granular cells. Phenotyping was further validated using mononuclear antibody staining (SWC1, SWC3a, MHC II, 2G6, CD2). Among the highly autofluorescent population, both small and large sized cells were observed. Animals infected with the bacterial respiratory pathogen *Pasteurella multocida* 880, associated with porcine pneumonia, showed a higher proportion of large cells compared to healthy controls where the ratio was inverted. Additionally, the number of low fluorescent cells decreased in infected BAL samples [84]. Using autofluorescence as a marker for alveolar macrophages from mice with chronic (CL13 variant) and acute (Armstrong variant) lymphocytic choriomeningitis virus infections in flow cytometry resulted in improved resolution of alveolar macrophages in fluorescent tdTomato reporter mice [85]. Mouse macrophages extracted from lung, spleen, liver and peritoneum exhibited autofluorescence in flow cytometry, although signals were significantly lower than those observed in macrophages obtained from human smokers [76].

##### 3.1.3.3 Identification

Only one study attempted to discriminate macrophages isolated from BAL from other immune cell types. Van Huizen et al. developed a deep learning model utilizing third harmonic generation and multiphoton‐excited autofluorescence. The model achieved good concordance with the known percentage of macrophages in the sample, performing better in comparison to its predictions for neutrophils, eosinophiles and lymphocytes [86].

#### 3.1.4 Microglia

Microglia are the macrophages of the central nervous system where they eliminate harmful substances, such as dead cells and microbes. They play a key role in neuroinflammation by releasing proinflammatory cytokines [87].

##### 3.1.4.1 Pathological States

Flow cytometry analysis (excitation/emission parameters were not reported) of microglia from old and young mouse brains identified a subpopulation of high autofluorescence microglia which were elevated in the old brains [88]. These represented approximately a third of microglia in the old mice and had increased hypertrophy and granularity, as well as altered lipid and iron content, phagocytosis and oxidative stress. Findings were consistent with the increased autofluorescence being due to the accumulation of lipofuscin, although this was not demonstrated directly [88]. Burns et al. found two subsets of microglia in mouse and non‐human primate brains differentiated by elevated (approximately 70%) and absent autofluorescence [89]. In the autofluorescent subset, the autofluorescence levels increased throughout adult life, although the proportion of cells which were autofluorescent did not change. Elevated autofluorescence levels were accompanied by increased size and complexity of lysosomal storage bodies [89] which were the primary sites responsible for the autofluorescence. The autofluorescent cells were also observed to have higher levels of LAMP1 and CD68, indicating an enlarged endolysosomal storage compartment. FACS was able to be used to separate autofluorescent and non‐autofluorescent fractions [89]. Subsequent proteomic assessment showed autofluorescent microglia had increased endolysosomal, autophagic, catabolic and mTOR‐related proteins.

In Burns et al.'s flow cytometry setup, autofluorescence was detected across multiple excitation/emission wavelength combinations, with the highest signals observed in the 660–735 nm emission range upon excitation with a 488 nm laser [89]. While microglial autofluorescence is generally associated with increased lipofuscin levels, the authors noted that although native fluorescence of lipofuscin is known to vary due to its heterogenous composition, the observed spectral properties of the microglia showed only partial overlap with the expected range of lipofuscin fluorescence. Additionally, autofluorescent microglia exhibited elevated ROS production a feature commonly linked to lipofuscin due to its incorporation of metal cations, but which could also indicate mitochondrial dysfunction.

Ritzel et al. found that depletion of brain microglia (using the colony stimulating factor 1 receptor agonist PLX5622) resulted in the loss of the autofluorescent subpopulation, which was not restored upon repopulation [88]. Burns et al. also investigated microglial depopulation via CSF1R agonism [89]. They found that autofluorescent microglia reappeared at a much slower rate than non‐autofluorescent microglia (4% of steady state levels compared to 67% at day 7). Furthermore, the fluorescence intensity of autofluorescent microglia post‐repopulation was markedly lower than pre‐depopulation. In Ritzel et al., it was shown that the loss of the autofluorescent subpopulation attenuated age‐related neurological deficits and neurodegeneration due to traumatic brain injury [88], supporting the conclusion that these highly autofluorescent microglia are a pathological subpopulation.

Lei et al. also investigated the dynamics of lipofuscin autofluorescence in microglia [55] using FACS in in vitro cultured immortalised cell lines. They demonstrated that feeding the cells with different rod outer segments and rod outer segment components (photoconduction elements of retinal cells) resulted in an increase in lipofuscin‐like autofluorescence. This increase was highest when using oxidised ROS (4‐hydroxynonenal treated) [55], supporting the contribution of oxidative stress to lipofuscingenesis in tissue with microglial cells.

#### 3.1.5 Dendritic Cells

Dendritic cells are specialised immune cells that play a major role in controlling adaptive immunity. Dendritic cells present antigen derived from self‐tissues and pathogens to T cells, subsequently directing the type of T cell response that ensues [90].

##### 3.1.5.1 Characterisation

Flow cytometry of dendritic cells from murine bone marrow had the highest NADH and FAD of all immune cell types assessed (CD4+ T cells, CD8+ T cells, B cells, macrophages, neutrophils), with values 8 times higher than adaptive immune cells [54]. Stimulation with 100 ng/mL LPS resulted in a minimal decrease in NADH fluorescence and no change on FAD levels. No significance was found using multiphoton microscopy, however, dendritic cells had significantly higher FAD expression than CD8+ T cells [54]. In two‐photon microscopy, dendritic cells from murine spleen had greater emissions (350–680 nm) at 710 nm excitation than T and B cells, but significantly lower emissions than macrophages [56]. This pattern was sustained at 750 nm, however at 800 nm the difference with T cells was lost. Fluorescence lifetimes of dendritic cells were significantly longer than for T cells, B cells and macrophages at 710 nm excitation. At 750 nm excitation, fluorescent lifetimes significantly differed only from macrophages, and at 800 nm, from T cells [56]. Activation of dendritic cells by Tnf‐α did not affect the average intensity of their emissions, however, fluorescence lifetime was significantly decreased [56].

##### 3.1.5.2 Pathological States

Cell death in murine spleen dendritic cells led to a decrease in NADH autofluorescence levels, while the FAD signal remained unaffected [54]. Gehlsen et al. found that in vitro assessment of dendritic cells resulted in significantly lower fluorescence intensities compared to in vivo measurements, noting that this effect was likely a result of applying higher excitation power to penetrate tissue [56].

#### 3.1.6 Mast Cells

Mast cells, present throughout the connective tissue, secrete vasoactive molecules that cause vasodilation and increase capillary permeability. They coordinate both innate and adaptive immune responses, to stimulate inflammation [91].

##### 3.1.6.1 Pathological States

Mast cells in deparaffinised angioimmunoblastic T‐cell lymphoma sections did not display observable autofluorescence [92]. Fluorescence microscopy utilised in a study of colonoscopic biopsies from patients with irritable bowel syndrome with tissue sections from urticaria pigmentosa, which are rich in mast cells, [93] similarly found mast cells were not autofluorescent. However, this study did not report the excitation/emission wavelengths applied making it challenging to compare these findings with other literature. In contrast, other research targeting the granules within mast cells, has identified specific patterns of autofluorescence: [94] imaged the secretion process of mucosal mast cells (cell line RBL‐2H3) using infrared three‐photon microscopy to excite serotonin. Using this technology, they were able to map the serotonin dynamic in stimulated mast cells, demonstrating that the overall kinetics of secretions followed the pattern of fast release from individual large granules accompanied by slower release from smaller granules and leakage of granule residues. In single photon microscopy of rat peritoneal mast cells, another study also found that stimulation resulted in autofluorescence changes due to serotonin, with a similar pattern of a large burst of activity followed by slow, sustained release [95]. In addition, different regions within cells had asynchronous activity, which the authors attributed to non‐uniform diffusion of the activating stimulant (polymyxin). Li et al. [96] used two‐photon microscopy to identify granules in mast cells, which contain a high concentration of tryptophan. Degranulation of in vitro cultured bone marrow derived mast cells, due to exposure to histamine liberator compound 40/80, could be observed as a sharp drop in tryptophan fluorescence. Mouse skin mast cells could also be visualised in vivo, indicating a subpopulation of dermal cells with bright, tryptophan autofluorescence that were present in C57Bl/6 mice, but lost in mast cell‐deficient Kit^*W‐sh/W‐sh*^ mice. This was further validated by an intradermal injection of bone marrow mast cells into the deficient mice to restore this population, further confirming their identity. Overall, the literature supports that mast cells do have autofluorescence in the UV range (or multiphoton equivalent) which can be used to give significant insights to their intracellular dynamics.

#### 3.1.7 Granulocytes

Granulocytes, or polymorphonuclear leukocytes, include neutrophils, eosinophils, and basophils. They are characterised by cytoplasmic granules filled with enzymes to aid in fighting invading microorganisms [97].

##### 3.1.7.1 Characterisation

In human granulocytes, agranulocytes, and platelets, peak emissions were observed at 350 nm (tryptophan) and 445 nm (NADH) when excited at 600 and 720 nm, respectively, using spectroscopic two‐photon microscopy [98, 99]. While the tryptophan channel showed uniform distribution in both quiescent granulocytes and agranulocytes, the NADH channel revealed structural differences, with granulocytes displaying distinct polymorphonuclear lobes [98, 99]. Similarly, mouse leukocytes also showed autofluorescence associated with tryptophan in two‐photon microscopy and very low signal for an NADH associated channel unevenly distributed within the granulocytes [100]. Spectrofluorometric analysis of human blood leukocytes found an emission peak at 340 nm for both granular and agranular cells when excited between 250 and 300 nm, likely attributed to tryptophan (excitation peak at 280 nm) [43]. For excitation wavelengths above 300 nm, both cell suspensions showed NADH associated autofluorescence with an emission peak at 440 nm and the highest signals measured upon excitation in the range of 350–360 nm [43]. Similarly, the excitation‐emission matrices of both human mono‐ and polymorphonuclear leukocytes showed the presence of FAD in addition to tryptophan and NAD(P)H [101]. Polymorphonuclear cells also displayed a peak at 500 nm excitation and 530 nm emission which could not be related to any fluorophores [101]. Granulocytes showed 1.4 to 2 times higher autofluorescence than monocytes and lymphocytes for excitation wavelengths above 265 nm. While granulocytes displayed some autofluorescence when excited with wavelengths less than 265 nm, the signal measured for agranulocyte was minimal [43]. Flow cytometry data also revealed that human granulocytes had approximately double the autofluorescence signal compared to monocytes for various excitation/emission combinations, though this ratio slightly changed with excitation at 488 nm and emission at 505–545 nm [46]. Activating polymorphonuclear cells (validated with OxyBURST Green) using *N*‐formyl‐methionyl‐leucyl‐phenylalanine (fMLP) increased NAD(P)H intensity 2.1 ± 0.5‐fold whereas no significant effect was observed for PMA stimulation [101]. Increased levels of tryptophan autofluorescence upon stimulation were linked to cell lysis [101]. The autofluorescence ratio of NAD(P)H and FAD in leukocytes, with intensities derived from spectral measurements, was distinct from that observed in the cervical cancer cell line HeLa [101].

##### 3.1.7.2 Pathological States

Flow cytometry of porcine BAL revealed a percentual increase of low‐autofluorescent granular cells in comparison to highly autofluorescent alveolar macrophages upon pneumonia infection [84]. In addition, two‐photon microscopy visualised healing processes of laser‐induced epithelial lesions in murine gut mucosa: Small heat‐induced bubbles (0.6–2.2 μm radius) only showed loss of autofluorescence [102]. With larger bubbles (*r* > 7 μm), autofluorescence loss in the affected cells was also observed, but within 1 min, autofluorescent polymorphonuclear leukocytes (eosinophils and neutrophils) accumulated at the site of injury [102]. Two‐photon imaging was also used to observe multilobed granulocytes and non‐autofluorescent lymphocytes in murine gut mucosa subjected to laser‐induced photodamage [103].

##### 3.1.7.3 Neutrophils

Neutrophils, a type of granulocyte, and also referred to as polymorphonuclear neutrophils (PMNs), are the most abundant immune cells in circulating blood in humans. They are the earliest responders to infections such as bacteria and fungi, migrating towards intruders and trigger an acute inflammatory response [104].

###### 3.1.7.3.1 Characterisation

Neutrophils originating from human bone marrow smears were described as exhibiting faintly yellow green autofluorescence under fluorescence microscopy [18]. Another source reported no autofluorescence under incident‐light fluorescence without specifying wavelengths [93], while others described it as weak [105, 106] with a few highly autofluorescent outliers [106] in spectrofluorimetry and FACS, respectively. In fluorescence microscopy, neutrophil autofluorescence did not correlate with the granules in a microfilariae assay [107] although slightly fluorescing granules were noted [43]. Peritoneal exudate and bone marrow polymorphonuclear neutrophils had similar emission spectra when excited at 361.1/363.8 nm in FACS, indicating an emission peak at 450–460 nm [19]. Two‐photon fluorescence spectroscopic imaging displayed an NADH related emission peak at 445 nm (excitation 700 nm) for neutrophils (identified by eGFP and DsRed) in zebrafish [108]. In the cytoplasm of human neutrophils, this NADH channel showed a uniform distribution, mirroring the absence of mitochondrial networks and the role of glycolysis as their major metabolic pathway [98]. The evenly distributed NAD(P)H associated autofluorescence within the cytoplasm was also observed with epifluorescence microscopy in [109]. Hafeman et al. [110] observed a decrease in NAD(P)H emission post‐inhibition of glucose‐dependent pyridine nucleotide reduction (2‐deoxyglucose) using flow cytometry. While neutrophils showed emission in fluorescence microscopy between 500 and 550 nm upon 488 nm excitation, no signal was detected for 638 nm excitation in the emission wavelength range of 660–740 nm [46].

Upon activation, a respiratory burst may occur in neutrophils which is defined as a rapid release of reactive oxygen species driven by NAD(P)H oxidase during phagocytosis [111]. This process has been associated with reduced autofluorescence across various stimulants, including PMA [109], sodium fluoride [109], *N*‐formyl‐methionyl‐leucyl‐phenylalanine (fMLP) [109, 112] and tumour necrosis factor (TNF) [109]. Stimulation with 12‐O‐tetradecanoyl phorbol‐13‐acetate (TPA) reduced the autofluorescence emission of activated neutrophils by 40%–80% [110]. Sodium dodecyl sulphate removed autofluorescent signals by releasing small molecules such as NAD(P)H from cells [109]. Phagosomes (red blood cells opsonised with rabbit anti‐sheep RBC IgG fraction) influenced autofluorescence, increasing it in close proximity and becoming punctate, with intensity quickly dropping upon interaction and oxidation [109]. Additionally, adherent neutrophils showed lower autofluorescence [109]. LPS (5 μg/mL) stimulation of murine neutrophils resulted in a minor increase of NADH and FAD in flow cytometry in NADH and FAD associated channels and no measurable change in multiphoton microscopy which the authors attributed to the inability of LPS to directly stimulate the NAD(P)H oxidase [54]. Higher LPS doses (50 μg/mL) significantly lowered human neutrophil autofluorescence compared to the quiescent state in confocal microscopy, though still higher than when stimulated with fMLP [112]. No significant change was observed for stimulation through synthesised peptidoglycan Pam3Csk4 [112].

Murine neutrophils emitted significantly less signal than dendritic cells in flow cytometry (2.8‐fold for excitation: 355 nm, emission: 425–475 nm [NADH], 2.1‐fold for 540–580 nm [FAD]) [54]. Also, human neutrophils exhibited less autofluorescence than eosinophils in two‐ and three‐photon microscopy [86]. Compared to highly autofluorescent eosinophils, they showed intermediate levels of autofluorescence similar to monocytes but higher than lymphocytes [43]. In cell line experiments, neutrophils (MoT), monocytes (THP‐1), and T cells (Jurkat) had less autofluorescence than eosinophils (HL 60) in two‐photon fluorescence microscopy [44]. Human neutrophils were more autofluorescent than monocytes at 436 nm excitation but less at 366 nm excitation, with emission intensities integrated from 490 to 680 nm and 430 to 620 nm, respectively [43]. The spectral emission curves showed no notable difference in shape upon either excitation wavelength [43].

Neutrophils showed low autofluorescence in comparison to eosinophils in FACS analysis [106, 107], with autofluorescence‐based FACS not affecting activation state, activation response or apoptosis rate [106]. Additionally, neutrophils had more narrow‐angle forward light scattering than eosinophils [107]. Autofluorescence was also used to distinguish neutrophils and CD4+ T cells in flow cytometry, with neutrophils expressing higher NADH signals than CD4+ T cells [54]. With fluorescence microscopy, neutrophils were not distinguishable from other subtypes (platelets, eosinophils, erythrophagocytic cells) whereas flow cytometry could detect a neutrophil population [46]. In fluorescence lifetime imaging, neutrophils showed longer mean fluorescence lifetimes than eosinophils and erythrophagocytic cells [46].

###### 3.1.7.3.2 Pathological States

Dead neutrophils had a significantly lowered optical redox ratio (NAD(P)H/[NAD(P)H + FAD]) in flow cytometry [54]. In confocal and epifluorescence microscopy of BAL from bacterial pneumonia infected mice, which mainly consisted of neutrophils (Ly6G), 40%–60% lowered autofluorescence was observed compared to BAL from healthy mice and ones with an LPS‐induced acute lung injury [112]. Additionally, different pneumonia types led to differences in polymorphonuclear neutrophil autofluorescence ( *Pseudomonas aeruginosa* pneumonia resulted in lower autofluorescence than *Staphylococcus aureus* pneumonia) [112]. In flow cytometry of BAL of hamsters with pancreatic elastase‐induced emphysema, 20% of the cells showed 4–5 times less autofluorescence and decreased forward and side scatter compared to the macrophage population, with 87% of these cells identified as polymorphonuclear neutrophils [83]. In mice infected with influenza A, using autofluorescence alongside immunostaining in spectral flow cytometry improved gating strategies and prevented neutrophils migration to other compartments [82]. Immune cell autofluorescence was detected using two‐photon imaging in murine models of airway inflammation and human tissue repair [113]. However, in this study eosinophils and neutrophils could not be differentiated [113]. In tissue samples from patients with angioimmunoblastic T‐cell lymphoma, fluorescence microscopy did not reveal any detectable autofluorescence in neutrophils and mast cells, unlike eosinophils, which showed strong emission [92].

In BAL of patients with ventilator‐associated pneumonia, polymorphonuclear neutrophil displayed 2.7‐fold less autofluorescence than healthy controls [112]. However, the sample size available was limited and unbalanced, including cells from 7 diseased and 2 healthy subjects [112]. Human neutrophils exposed to *E. coli* bacteria showed a red‐shift in their NADH associated channel in two‐photon microscopy, which was related to an increase in their free‐to‐bound NADH ratio [98].

In summary, although many studies have reported different autofluorescence levels for neutrophils and eosinophils, there are instances in which the two immune cell populations were indistinguishable based on their native fluorescence.

###### 3.1.7.3.3 Identification

FACS sorting of neutrophils versus eosinophils based on their autofluorescence intensity resulted in purity of over 99.5% for human blood neutrophils and murine bone marrow neutrophils, confirmed by cell morphology and CD16+, CD49d markers [106]. In addition, using 0° and 90° light scatter with an autofluorescence parameter improved FACS enrichment of neutrophils by 5%, reaching over 95% [19].

BAL samples of pneumonia‐infected mice, which consisted of approximately 69% neutrophils compared to 5% in control samples, could be distinguished from healthy controls with an AUC of 0.89 ± 0.01 [112]. A multimodal approach using third harmonic generation and two‐photon autofluorescence microscopy combined with a deep learning regression network (ResNet50), identified eosinophils, neutrophils, lymphocytes, and macrophages in blood and BAL fluids from both healthy individuals and interstitial lung disease patients [86]. The model had a validation mean absolute error of 13.6%, typically underestimating neutrophil fractions unless they were present in small numbers [86].

##### 3.1.7.4 Eosinophils

Eosinophils, another type of granulocyte, are produced in the bone marrow and released into the blood stream, where they play a key role in fighting parasitic infections and mediating allergic reactions. Additionally, they contribute to immune responses against bacteria, viruses, and tumour cells [42].

###### 3.1.7.4.1 Characterisation

Eosinophil granules from human bone marrow smears exhibited intense autofluorescence in fluorescence microscopy with all eosinophils from one patient displaying similar levels of autofluorescence [18]. This autofluorescence originated from the granules within the cytoplasm [43, 93, 107], as confirmed by adherence assays to microfilariae [107]. Fuerst et al. [18] described the eosinophil autofluorescence as intense yellow, whereas Weil et al. [107] and Mayenco et al. [114] found an emission peak at 520 nm (green) and excitation maxima at 370 nm (380 nm) and 450 nm in eosinophil granules from blood of healthy and eosinophilia patients. The autofluorescence primarily stemmed from FAD (> 85%), FMN, and riboflavin according to Mayeno [114]. Van Huizen et al. [86] observed increased signals in the cytoplasm using 3‐photon (emission: 380–420 nm) compared to 2‐photon (emission: 562–665 nm) autofluorescence microscopy at 1070 nm excitation, attributing the signal to NADH though their measurement notably also overlaps with flavins autofluorescence. Barnes et al. [105] identified three excitation/emission maxima of live eosinophils extracted from human blood: 280/330 nm (tryptophan), 360/440 nm, and 380/415 nm, with no significant signal above 450 nm excitation contradicting [107]. Monici et al. reported bright autofluorescence from human blood granules in the cytoplasm using fluorescence microscopy [43]. Isolated eosinophiles emitted blue/violet light upon 365 nm excitation, while eosinophils in tissue emitted amber‐gold [105].

Eosinophils showed higher emission [105, 106, 107, 114] and less narrow‐angle forward light scatter (2°–15°) compared to neutrophils [107]. Eosinophils exhibited the brightest autofluorescence in microspectrofluorometric analysis compared to neutrophils (intermediate), monocytes (intermediate), and lymphocytes (lowest), attributed to differences in cell metabolism [43]. These differences were also pronounced in their emission spectra upon 366 nm and 436 nm excitation [43]. Similarly, an eosinophil cell line (HL 60) expressed higher autofluorescence than neutrophils (MoT), T cells (Jurkat), monocytes (THP‐1) and epithelial cells (KB) in two‐photon fluorescence microscopy (excitation: 700–900 nm, emission: 500–600 nm) [44]. In fluorescence microscopy, eosinophils and erythrophages showed emission (660–740 nm) upon 638 nm excitation where neutrophils did not show any signal [46]. In fluorescence lifetime imaging, eosinophils had shorter mean fluorescence lifetimes than neutrophils but higher lifetimes than erythrophagocytic cells [46]. In flow cytometry, eosinophils displayed high signals in the FITC and ECD channels and were more autofluorescent than erythrophagocytic cells for excitation/emission 405 nm/427.5–472.5 nm and 488 nm/505–545 nm [46].

###### 3.1.7.4.2 Pathological States

Autofluorescence levels for eosinophils from various diseased donors (e.g., from cancer) could not be correlated to patient disease or drug therapy when estimated and grouped visually [18]. However, eosinophils from patients with eosinophilia showed different autofluorescent properties compared to healthy blood donors, with an inverse relationship between eosinophil counts and their autofluorescence: lower counts were linked to higher fluorescence [107].

Immune cell autofluorescence was observed ex vivo in murine models of airway inflammation and human tissue repair using multiphoton microscopy, but eosinophils and neutrophils could not be distinguished [113]. In tissue probes of patients with angioimmunoblastic T‐cell lymphoma, fluorescence microscopy showed high autofluorescence emission for polymorphonuclear granulocytes identified as eosinophils through Giemsa staining while singular mast cells and neutrophils did not exhibit autofluorescence [92]. Similarly, eosinophils showed autofluorescence in active inflammation in patient colonoscopy biopsies while neutrophils, mast cells, and lymphatic cells displayed no signal in incident‐light fluorescence [93]. This was utilised to stage eosinophilia and also had ability of indicating disease type: for example, Crohn's disease patients had 57% autofluorescent eosinophils while for ulcerative colitis only 9% were emitting signal [93]. In approximately 30% of fixed bone marrow biopsies of diseased patients (leukemia, eosinophilia), autofluorescence displayed eosinophil degranulation using epifluorescence microscopy [115].

###### 3.1.7.4.3 Identification

In FACS, eosinophils could be distinguished from neutrophils based on their emission and scatter achieving purities above 98%, though with an average yield of 65% [107]. Additionally, eosinophils (CD16−, CD49d+) could be separated from neutrophils using various excitation/emission combinations, resulting in enrichments of 97% but limited yield [106].

In a multimodal approach combining third harmonic generation and two‐photon autofluorescence microscopy, a deep learning regression network based on a pretrained neural network (ResNet50) identified fractions of eosinophils, neutrophils, lymphocytes and (when applicable) macrophages in blood and BAL fluids from healthy and interstitial lung disease patients, with a validation mean absolute error of 13.6% [86]. Eosinophils were underestimated in BAL samples [86].

In nasal smears from patients suffering from rhinitis, eosinophils could be distinguished from epithelial cells through their higher autofluorescence achieving an accuracy of 97%, a sensitivity of 100%, specificity of 98% and AUC of 98% [44]. Additionally considering cell size as a morphological feature improved sensitivity, specificity, positive predictive value, negative predictive value and accuracy to 100% [44].

#### 3.1.8 Erythrophagocytic Cells

Erythrophagocytic cells, are phagocytic cells including macrophages, neutrophils and monocytes, that have ingested aged or damaged erythrocytes [116].

##### 3.1.8.1 Pathological States

To induce erythrophagocytosis, human leukoconcentrates were mixed and incubated with blood samples from a different donor [46]. Under fluorescence microscopy, the erythrophagocytic cells and eosinophils exhibited emission signals (660–740 nm) upon 638 nm excitation, whereas neutrophils showed no emission [46]. The autofluorescence in erythrophagocytic cells originated from the site ingested erythrocyte and was therefore related to degradation products like bilirubin [46]. Additionally, fluorescence lifetime imaging revealed that erythrophagocytic cells had a fluorescence lifetime of 0.7 ± 0.1 ns, which was higher than that of neutrophils but lower than that of eosinophils. In flow cytometry, erythrophagocytic cells also displayed high forward scattering indicative of phagocytosis and a red‐shifted signal which could be used for effective gating [46].

#### 3.1.9 Natural Killer Cells

Natural killer (NK) cells are innate lymphocytes that target and destroy pathogens and tumour cells. Though primarily innate, they also feature characteristics of adaptive immunity and can form an immunological memory [117].

##### 3.1.9.1 Characterisation

Only one study [118] investigated autofluorescent characteristics of natural killer cells. Using multiphoton fluorescence microscopy, they showed that in vitro activated primary human NK cells (treated for 24 h using IL‐12, IL‐15 and IL‐18) expressing CD69 had a significantly higher optical redox ratio, increased fraction of unbound NAD(P)H, and longer FAD mean fluorescence lifetime, while NAD(P)H mean fluorescence lifetime decreased compared to quiescent, CD69‐negative control cells.

##### 3.1.9.2 Identification

In a random forest classifier (for separating CD69^+^ activated from CD69^−^ controls), the highest weighted parameters were control‐normalised optical redox ratio (20.45%), unbound fraction of NAD(P)H (20.15), protein‐bound NAD(P)H fluorescence lifetime (17.45%), and unbound fraction of NAD(P)H fluorescence lifetime (13.35%). Activated human peripheral blood NK cells could be discriminated from quiescent NK cells with 92.6% accuracy, while NK cells could be discriminated from other cell types (B and T cell) with 98.9% accuracy using optical metabolic imaging and a random forest classifier. When classification was expanded to include discrimination of activated and quiescent cell types, activated NK cells could be discriminated from activated B and T cells as well as quiescent B, NK and T cells with 95.6% accuracy, while quiescent NK cells could be discriminated with 94.9% accuracy [118].

### 3.2 Adaptive Immunity

Adaptive immunity is a highly specialised and targeted immune response that provides long‐term protection by recognizing and responding to specific pathogens through the actions of T and B lymphocytes (Table 2) [119].

#### 3.2.1 Lymphocytes

Lymphocytes, present in lymph nodes, lymphoid organs, and peripheral blood, recognise and respond to specific antigens. The primary types of lymphocytes are B cells, which produce antibodies, and T cells, which regulate immune responses and directly attack infected cells [51].

##### 3.2.1.1 Characterisation

In fluorescence imaging, the autofluorescence of human lymphocytes displayed a homogeneous cytoplasmic distribution [43]. Their emitted autofluorescence was the lowest in intensity compared to eosinophils (highest), neutrophils (intermediate), and monocytes (intermediate) using microspectrofluorometric analysis [43]. The emission spectra of lymphocytes and eosinophils differed under 366 ± 10 nm and 436 ± 10 nm excitation (emission: 430–620 nm, 490–680 nm, respectively), whereas the spectral differences between lymphocytes, neutrophils, and monocytes were minimal [43]. Fixed mouse peripheral lymphocytes exhibited higher autofluorescence levels than live ones for UV, violet, and blue excitation in spectral flow cytometry [85]. Using flow cytometry, human lymphocytes could be distinguished from monocytes and granulocytes [46].

Exposing PBMCs to medicinal plants extracts (*Ageratum fastigiatum*, *Eriosema campestre*, *Pseudobrickellia brasiliensis*), showed a treatment and concentration dependent increase in autofluorescence [120]. In addition, treatment increased the lymphocyte population to 91.3%, with 82.3% being HLA‐DR positive, indicating activation (untreated: 22.1%) [120].

##### 3.2.1.2 Pathological States

In neurodegenerative diseases, lymphocytes from patient buffy coats, confirmed by electron microscopy, displayed inclusions visible through autofluorescence on an epifluorescence microscope (excitation < 500 nm, emission > 515 nm) [121]. While lymphocytes without inclusions were non‐autofluorescent, those containing cytoplasmic granules (diameter 1–2 μm), associated with lipofuscin accumulations in conditions such as neuronal ceroid‐lipofuscinoses and Hallervorden‐Spatz syndrome, all showed a yellow‐orange signal. Lymphocytes with parallel tubular arrays exhibited a widespread, blurry green autofluorescence, linked to an unknown diagnoses or Leber congenital amaurosis [121]. In two‐photon microscopy of heat‐damaged murine gut mucosa tissue, lymphocytes appeared dark due to a predominantly non‐autofluorescent nucleus surrounded by a thin layer of cytoplasm [102]. Similar results were observed for broad band excitation and a tuneable laser, facilitating identification combined with the lymphocyte size and speed [103]. In the ocular conjunctiva‐associated lymphoid tissue (CALT) of mice stimulated with *Chlamydia trachomatis* serovar C or a combination of ovalbumin and cholera toxin B, distinct lymphocytes were observed using two‐photon microscopy in a 3D image stack up to 65 μm tissue depth [122]. These lymphocytes appeared as dark nuclei surrounded by a small autofluorescent cytoplasmic rim, aggregated in lymphoid follicles [122]. Similarly, in multispectral fluorescence imaging microscopy, the relatively large non‐autofluorescent nuclei in human lymphocytes compared to the autofluorescent cytoplasm resulted in weak signals for cells from excised, cryopreserved lymph node tissue of patients with lymphadenopathy (3x reactive hyperplasia, 2x Hodgkin's disease) [123]. Spectral analysis revealed no differences between the autofluorescence of lymphocytes from lymphoid follicles and lymphoma cells, though this may differ for live tissue [123].

Lymphocyte migration in response to M cells, which sample gut antigens and transport them from the luminal surface to the sub‐epithelial tissue, was visualised using two‐photon microscopy in murine Peyer's patches of the small intestine. Migration speeds of up to 8.2 μm/min were observed [124].

##### 3.2.1.3 Identification

A deep learning regression network based on a pretrained neural network (ResNet50), utilizing multimodal data from third harmonic generation and two‐photon autofluorescence microscopy (excitation: 1070 nm, emission: 562–665 nm), could identify fractions of eosinophils, neutrophils, lymphocytes, and macrophages, with a validation mean absolute error of 13.6% [86].

##### 3.2.1.4 B Cells

B cells are primarily responsible for producing antibodies to target specific antigens. Additionally, they participate in antigen presentation to T cells and cytokines secretion [125].

###### 3.2.1.4.1 Characterisation

Flow cytometry of murine spleen B cells showed that they had significantly lower NADH and FAD fluorescence than dendritic cells but did not differ from CD4+ or CD8+ T cells [54]. Stimulation of these cells (IL‐4, LPS, α‐IgM) increased NADH 3.7‐fold with similar effects for FAD. When assessing this by multiphoton microscopy, the change was not significant. However, in another study that applied optical metabolic imaging to human peripheral blood B cells, it was shown that activated B cells (anti‐CD40 antibody, IL‐4) had a higher redox ratio (NAD(P)H/[NAD(P)H + FAD]) than quiescent ones [118]. Murine spleen B cells were assessed by two‐photon microscopy and compared to T cells, dendritic cells and macrophages in Gehlsen et al. [56]: At 710 nm excitation, B cells displayed significantly higher emission intensities compared to T cells, while their emission intensities were lower than those of dendritic cells and macrophages. This effect persisted for dendrites and macrophages at 750 and 800 nm but was not apparent for T cells. With regards to fluorescence lifetimes, B cells were shorter than T cells, dendritic cells and macrophages at 710 nm, longer than macrophages at 750 nm, and shorter than T cells at 800 nm. Activation of B cells (LPS) resulted in significantly increased fluorescence intensity at 750 nm and significantly decreased fluorescence lifetime [56].

###### 3.2.1.4.2 Pathological States

Cell death (indicated by Sytox stain) decreased NADH in B cell, but did not affect FAD, resulting in a significant elevation of the redox ratio (NADH/[NADH + FAD]) [54]. In vitro cultured B lymphoma cells (malignant B cells, CA46 cell line) had distinct autofluorescent spectra with lower fluorescence compared to quiescent T cells (from human buffy coat) when excited at 351 nm using confocal microscopy, but higher autofluorescence than T cells (quiescent and stimulated, anti CD3 and anti CD28) upon 458 and 488 nm excitation [126]. At injection into murine eyes, the location of the B lymphoma cells was able to be elucidated with autofluorescence at 488 nm.

###### 3.2.1.4.3 Identification

Activated human peripheral blood B cells could be discriminated from quiescent B cells with 93.4% accuracy, while B cells could be discriminated from other cell types (NK and T cell) with 98.5% accuracy using optical metabolic imaging and a random forest classifier [118]. When classification was expanded to include both activation status and cell type, activated B cells were distinguished from active NK and T cells as well as quiescent B, NK and T cells with an accuracy of 96.5%. In this comparison, quiescent B cells were classified with a similarly high accuracy of 96.2% [118].

##### 3.2.1.5 T Cells

Cell‐mediated immunity, activated when pathogens invade cells, is primarily regulated by T lymphocytes, which recruit and activate phagocytes and directly attack infected host cells. The key subtypes of pan (CD3+) T cells are CD4+ helper T cells, which are responsible for B cell and macrophage activation, and stimulation of inflammation; CD8+ cytotoxic T cells which target and eliminate infected cells; and regulatory T cells (Tregs), which help prevent the excessive immune responses [51].

###### 3.2.1.5.1 Characterisation

Walsh et al. [127] utilised two‐photon autofluorescence imaging microscopy to assess the intensity and fluorescence lifetime of NAD(P)H and FAD of quiescent and stimulated human pan‐T cells (24 and 48 h of CD2, CD3, and CD28 antibody exposure). For stimulated cells, there was a significant increase in parameters associated with heightened metabolic rates. These included cell size and metabolic indicators such as optical redox ratio (NAD(P)H/[NAD(P)H + FAD]), mean fluorescent lifetime of NAD(P)H, and the respective free fractions of NAD(P)H and FAD [127]. Activated T cells showed a lower redox ratio (NAD(P)H/[NAD(P)H + FAD]) in their cytoplasm compared to mitochondrial areas [128]. Although cell size varied among the six participating donors, autofluorescent properties remained consistent across all donors and in repeated blood draws from the same donor [127]. In the first 10 min post‐stimulant exposure, increased levels of NAD(P)H were observed in the nucleus, whereas the cytoplasm displayed heightened signatures between approximately minutes 6–9 [127]. A 24‐h redox ratio measurement post stimulation showed an initial drop between 0.5 and 1 h, before elevated levels were measured after 2 h. While Walsh et al. [127] measured no significant differences for the mean lifetime of FAD between 24 and 48 h antibody exposure compared to resting pan‐T cells and CD8+ T cells, Pantanelli et al. [126] reported a significant increase of autofluorescence for pan‐T cells only after 72 h with insignificant changes after 1 day of stimulation (CD3, CD28 antibodies). Co‐culturing human CD4+ and CD8+ T cells increased the proportion of free NAD(P)H in both quiescent and activated CD8+ T cells compared to their isolated state [127]. Memory CD8+ T cells displayed significantly lower NAD(P)H mean lifetimes than naïve CD8+ T cells [127]. A significant decrease of the mean fluorescent lifetime of FAD after activation was only observed for the pan‐T cell population and not for isolated CD8+ T cells indicating an effect of culture conditions [127]. Heightened autofluorescence intensities and lifetimes for stimulated (CD3, CD28) pan‐T cells from murine spleen were also reported by Gehlsen et al. [56] using two‐photon microscopy. Similarly, stimulating murine CD8+ T cells in plates pre‐coated with antibodies (α‐CD3/α‐CD28, 10 μg/mL) resulted in a significant increase in NADH levels (4.5‐fold by flow cytometry) [54]. However, this increase was not as high as observed for equally stimulated murine CD4+ T cells (6‐fold increase using flow cytometry) [54]. Heightened NADH and FAD signals for stimulated murine CD4+ and CD8+ T cells were also observed using multiphoton microscopy, particularly for CD4+ with a 1.9‐fold increase for NADH [54]. Additional tests using metabolic inhibitors linked the elevated NAD(P)H and redox ratios (NAD(P)H/[NAD(P)H + FAD]) in activated T cells to heightened cell metabolism essential for cell replication and differentiation [118, 127, 128]. Notably, the fluorescence lifetime and intensity redox ratios in T cells showed limited correlation, with fluorescence lifetime redox ratios which had lower variability [128].

In flow cytometry, autofluorescence could be used to gate CD4+ T cells and neutrophils based on respective low and high NADH signals [54]. Pan‐T cells from mice spleen exhibited significantly lower autofluorescence intensities compared to dendritic cells and macrophages for two‐photon excitation of 710, 750, and 800 nm, and lower emission intensities than B cells upon 710 nm excitation [56]. In addition, T cells showed significantly lower fluorescence lifetimes compared to B cells and macrophages upon 710 nm excitation, and higher lifetimes compared to dendritic cells [56]. At 800 nm, T cells had significantly higher fluorescence lifetimes than B cells, dendritic cells, and macrophages [56]. Immortalised T cells (Jurkat) had less autofluorescence than eosinophils (HL 60) in two‐photon fluorescence microscopy [44].

Long term culture of primary human T cells, with multiple PHA stimulation rounds and IL‐2 exposure, resulted in an age‐relate lipofuscin accumulation in the cells. This caused a significant increase in autofluorescence after 23 weeks of in vitro culture [129].

###### 3.2.1.5.2 Pathological States

T cell death via autophagy correlated with changes in lipofuscin, resulting in reduced autofluorescence levels [129]. Dead quiescent murine CD4+ and CD8+ T cells (indicated by Sytox stain) exhibited significantly decreased NADH levels but unchanged FAD signals, resulting in a significantly decreased redox ratio (NAD(P)H/[NAD(P)H + FAD]) [54].

Pantanelli et al. demonstrated in their in vitro experiments that primary quiescent T cells emitted greater autofluorescence than B lymphoma (malignant B cells, CA46) when excited at 351 nm. Both resting and activated T cells displayed lower autofluorescence than B lymphoma when excited at 458 and 488 nm, with a slightly more pronounced difference at 458 nm. Additionally, ex vivo measurements showed visible autofluorescence at 488 nm excitation from both B lymphoma and T cells after injection into a mouse eye [126]. In freshly excised lymph nodes of C57Bl/6 FoxP3‐EGFP mice with large tumours (250–430 mm^3^ B16F0 melanoma, 14–15 tumour growth period), T cells showed significantly increased NAD(P)H mean fluorescence lifetimes compared to T cells from tumour‐free mice and those with small tumours (40–250 mm^3^, 10–11 days growth period) due to prolonged lifetimes of protein‐bound NAD(P)H [130]. This increase was specifically linked to bound NADPH biosynthetic processes which, however, could only be consistently recorded in 23%–83% of cells per lymph node sample. Both CD4+ T‐helper and CD8+ T‐cytotoxic cells from mice with large tumours expressed slightly elevated activation (CD69) and proliferative (IFN‐γ, Ki67) markers compared to mice with small tumours, indicating metabolic changes. However, the activation marker CD25 did not differ significantly between the three groups (control, small tumour, large tumour) and no significant differences among the three cohorts were detectable solely based on autofluorescence intensity [130]. A consecutive study investigated autofluorescent changes in T cell responses as a potential marker for predicting effectiveness of immunotherapy in a melanoma mouse model. The amplitude ratio of free NADH to protein‐bound NAD(P)H was significantly higher for immunotherapy responders (anti‐CTLA‐4‐therapy, multiple treatments) compared to non‐responders and an untreated tumour control group [131]. This increase of free and reduction of protein‐bound NAD(P)H might indicate a metabolic shift towards glycolysis [131]. Responders and non‐responders in this study were verified through tumour size, activation, and proliferation measurements. No significant increase of either protein‐bound NAD(P)H lifetime or phosphorylated NADPH amplitude were observed for effective tumour treatments unlike in the study on large tumours versus healthy/small tumours [131].

###### 3.2.1.5.3 Identification

Activated and quiescent T cells, drug‐responsive and drug‐resistant breast cancer cells, as well as their mixture ratios were simulated from autofluorescence datasets from prior studies (T cell data [127]; breast cancer data from [132]). Classification of the simulated data achieved an accuracy of 92% [133].

For classifying activation status of pan‐T cells, the features with the highest weights were free NAD(P)H, cell size and optical redox ratio [127]. For CD8+ T cells, the most important features were the free fraction of NAD(P)H, optical redox ratio, and mean fluorescent lifetime of NAD(P)H. Using UMAP and a logistic regression model, the AUC was 0.975 for pan‐T cells and 0.996 for isolated CD8+ T cells including all autofluorescence features from NAD(P)H and FAD. The AUC slightly decreased to 0.965 and 0.994 when using only NAD(P)H related features [127]. Evaluating Walsh et al.'s T cell data with CNN transfer‐learning resulted in an average accuracy of 93.56% [134]. Similar results with an AUC of 0.92 for quiescent and activated (72‐h exposure to CD2/CD3/CD28) T cells were achieved with a more cost efficient device although the study involved less patients [135]. When [118] classified B cells, natural killer cells and Walsh et al.'s T cells, the T cell population was identified with 97.8% accuracy. Additionally classifying for cell activation reduced accuracy to 90% with an AUC of 0.97.

### 3.3 Uncategorised Immune Cells

This section on uncategorised immune cells includes studies that focus on immune responses without specifying cell types or findings based on mixed immune cell populations (Table 3).

#### 3.3.1 Characterisation

The autofluorescence properties of human leukocytes were investigated by Yakimov et al. using fluorescence lifetime imaging [46]. While they detected no signal in their NAD(P)H associated channel, their FAD channel showed autofluorescence. In epifluorescence imaging of blood vessels, both the excitation light and emitted autofluorescence signals of leukocytes were absorbed by erythrocytes [46].

Flow cytometry analysis of unstimulated murine dendritic cells, neutrophils, macrophages, B cells and T cells (CD4+, CD8+), showed that innate immune cells exhibited more autofluorescence than adaptive cells at 355 nm excitation [54]. This difference was observed in both the NADH‐ and FAD‐associated channel [54]. Similar results were obtained using multiphoton microscopy at 810 nm excitation, with innate cells displaying 1.6‐fold intensity in the NADH channel and 1.7‐fold intensity in the FAD channel compared to adaptive immune cells [54]. T‐distributed stochastic neighbour embedding (t‐SNE) analysis of autofluorescence from epifluorescence microscopy and flow cytometry revealed distinct clusters of erythrophagocytic cells, eosinophils, lymphocytes, neutrophils and platelets [46]. Leukocytes (polarised RAW264.7 macrophage, human neutrophils and monocytes) exhibited stimulant‐dependent changes in both the amplitude and frequency of NAD(P)H‐associated autofluorescence [136]. Specifically, stimulation with IFN‐γ tripled amplitudes, while combining IFN‐γ with IL‐6 or IL‐2 doubled the frequency [136]. Other stimulation conditions tested included IL‐6, IL‐2, IL‐1β, IFN‐γ + IL‐1β, TNF‐α, and IFN‐γ + TNF‐α [136].

#### 3.3.2 Pathological States

Flow cytometry analysis of autofluorescence spectra using opt‐SNE revealed that leukocyte samples from mice on different diets had similar populations while those from mice with chronic (lymphocytic choriomeningitis virus CL13) and acute (Armstrong) viral infections were markedly different, despite having similar spectra [85]. Chang et al. found that lymph node leukocytes of healthy mice showed low autofluorescence in spectral flow cytometry, while BAL leukocytes exhibited high signal [82]. The intensities of lung tissue leukocytes were lower than for BAL leukocytes in the red region and they emitted broad autofluorescence in the UV and violet channels. For pneumonia infected mice (Influenza A), the autofluorescence spectra of leukocytes from BAL and lung tissue changed over time, in contrast to lymph node leukocytes. In addition, the spectral profiles differed upon inflammation type (lipopolysaccharide, house dust mite) [82].

Additionally, autofluorescence was used for observing immune cell migration patterns. In two‐photon microscopy, cell autofluorescence facilitated tracking leukocyte the vascular system and visualised inflammation in mice, including high LPS‐induced and mild UV‐induced inflammation [100]. Furthermore, intravital two‐photon microscopy facilitated visualisation of immune cell migration in mice cornea towards inflammation sites (sutures) characterising their behaviour and velocities through cytoplasmic autofluorescence [137]. Explanted inflamed tissue of murine bleomycin induced pulmonary fibrosis demonstrated a decrease in NADH and increase in FAD in multiphoton microscopy, which correlated highly with disease severity measured by the Ashcroft score [138]. Using autofluorescence, inflammation in murine pulmonary fibrosis was detected with an AUC of 0.92 (accuracy 0.81, Matthews correlation coefficient MCC 0.66) [138]. When evaluating disease severity, a Kappa value of 0.21 was obtained (F1 0.43, MCC 0.26). These results were further improved by employing a multimodal approach that integrated second harmonic generation and Raman spectra, respectively [138].

In the context of cancer research, Heaton et al. investigated autofluorescence signals of spleen immune cells and B78 melanoma tumour cells in two mouse models [139]. Utilising CD4 mCherry labelling (which did not overlap with autofluorescence channels), T cells (CD4+, CD4 + CD8+), and NKT cells were identified with small amounts of other subsets such as macrophages, dendritic cells, neutrophils, NK cells, and B cells. FLIM captured NAD(P)H and FAD autofluorescence intra‐vitally (bleed through of other native fluorophores acknowledged), while second harmonic generation was used to measure collagen. The authors found that tumour infiltrating immune cells were larger and expressed reduced mean FAD and NAD(P)H fluorescence lifetimes, potentially related to altered tumour environments. Additionally, autofluorescence differences were noted between the two mouse models [139]. Another study by Yang et al. [65] assessed the autofluorescence of immune cells in response to immunotherapy in a mouse model of triple‐negative breast cancer through in vivo imaging. The authors found that FAD autofluorescence primarily highlighted a small subset of immunosuppressive cells, including M2 macrophages (CD206^+^CD11b^+^CD301b^+^) and regulatory T cells (Tregs, CD4^+^CD25^+^). High NAD(P)H autofluorescence was emitted by cancer cells and immunoactive cells including dendritic cells (CD11c^+^), M1 macrophages (F4/80^+^MCP1^+^CCR2^+^), and Th cells (CD4^+^) [65].

## 4 Conclusion

Autofluorescence properties of a variety of immune cells have been investigated covering both innate (monocytes, macrophages, microglia, dendritic cells, mast cells, neutrophils, eosinophils, natural killer cells) and adaptive immunity (B cells, T cells). In addition, research on polarisation of macrophages (M0, M1, M2a, M2b, M2c and M2d) [72] and CD4+ T cells, CD8+ T cells and regulatory T cells has been reported [54, 127]. Noticeably, none of the included work has investigated basophil autofluorescence though murine basophils have been previously described as somewhat autofluorescent [140] and NAD(P)H autofluorescence of the basophilic leukemia cell line RBL‐2H3 has been explored [141]. Although basophils comprise the minority of granulocytes, they play a crucial role in mediating allergic reactions and inflammatory responses [142]. In addition, autofluorescent differences between many immune cell subtypes, for example, classical and non‐classical monocytes, have not been investigated.

Accurate reporting of excitation and emission wavelengths is critical in autofluorescence research, as they can indicate specific native fluorophores, allowing researchers to connect measured signals to underlying biological processes. The identified autofluorophores in the reviewed studies include flavins (FAD, FMN, riboflavin), NAD(P)H, haemoglobin, bilirubin, lipofuscin and tryptophan. These fluorophores reflect key cellular processes: flavins and NAD(P)H are involved in cellular metabolism, particularly in redox reactions [6]. Haemoglobin plays a significant role in oxygen transport, while bilirubin is the final product of heme metabolism [143]. Lipofuscin, accumulating in aging non‐dividing cells due to inefficient recycling of damaged components, is linked to cellular dysfunction [6]. Tryptophan, an amino acid, acts as an immune modulator and aids in tissue homeostasis restoration [144]. An overview of native fluorophores and their properties is given in [6].

While many studies provided thorough reporting of wavelength ranges, this review identified some inconsistencies in the methods section of several reports: two studies omitted wavelength ranges [93], one described emission using vague colour description [84], and several studies required referencing related works or converting filter names to wavelength data, which can be challenging for decommissioned equipment [17, 77, 92]. These inconsistencies not only obstruct the ability to link measured autofluorescence to biological processes but also complicate comparisons across studies.

Research has primarily focused on characterising immune cell autofluorescence and examining signal changes in various diseases. Regarding characterisation, there is a general agreement that macrophages and dendritic cells express the highest autofluorescence intensities, although the ranking between these two cell types varies between studies [54, 56]. Among granulocytes, eosinophils consistently show higher autofluorescence intensities than neutrophils [43, 44, 46, 105, 106, 107, 114, 145]. Monocytes and lymphocytes (B and T cells) display the lowest levels of autofluorescence [43, 44, 46, 54, 56, 76], which may be related to their large nucleus‐to‐cytoplasm ratio as the nucleus generally emits low signals. No comparisons of autofluorescence levels were made for microglia, and natural killer cells.

In terms of pathological conditions, studies investigated a wide range of diseases such as pneumonia [112], inflammation [68], and cancer [130]. The response of immune cells to various stimuli can either increase or decrease autofluorescence, providing insights into infections, such as detecting pneumonia in ventilated patients through neutrophil autofluorescence changes when compared to healthy controls [112]. A wide range of applications for immune cell autofluorescence has been explored. Numerous studies assess immune cell changes within tumor microenvironments, analysing how their signals alter in response to cancer cells or malignant transformations. These studies often focus on immune cell types such as monocytes [50], macrophages [50, 59, 64], eosinophils [92], B cells [126], and T cells [126, 130, 131], to understand their roles and behaviors in cancer. In addition, healing process have been studied for both macrophages [69] and granulocytes [102] and the effects of atherosclerosis on cellular autofluorescence have been investigated for monocytes [48, 49] and macrophages [48, 67].

Most research has been conducted in cell cultures, with relatively few studies focusing on tissue‐level immune cell autofluorescence. Tissue‐based studies face challenges due to light absorption of overlying tissue layers and interference from erythrocytes in the blood vessels. However, there have been successful applications, for example, in monitoring immune cell migration [100]. Since PBMCs are easily obtained from blood, the focus on cell culture studies does not pose a significant barrier to the clinical transferability of these findings, unlike other applications of cellular autofluorescence, such as investigating its potential in assessing oocyte quality [146] or for melanoma diagnosis [146, 147].

Despite promising research on the applicability of cellular autofluorescence for immune cell identification, activation status, and quantitative assessment of pathological changes remains limited though is a focus of recent studies. Building reliable models requires vast datasets. Autofluorescence in immune cell has been detected through intensity, fluorescent lifetime, or oscillation frequence and amplitude, using a variety of fluorescent microscopes, spectrofluorometers, and flow cytometry or FACS devices. While flow cytometry offers for high‐throughput cell analysis, it cannot capture morphology and texture features. Imaging flow cytometers, such as the Cytek Amnis ImageStreamX Mk II Imaging Flow Cytometer [148], offer a potential solution, though its current limitation to 12 channels restricts its utility. Vast datasets can also be generated using high‐content imaging systems, such as the Operetta system by Revvity, thereby enabling comprehensive analysis of cellular dynamics and phenotypic changes [149].

Immune cell autofluorescence offers the potential to eliminate the need for laborious staining procedures, reducing both costs and processing time while potentially improving accuracy. Additionally, it could allow for further downstream processing of cells post‐analysis, depending on the measuring device used. Studies by Streck et al. [76] and Soethout et al. [81] highlight the advantages of autofluorescence over conventional immune cell staining for macrophages, which express high autofluorescence levels that complicate staining and increase the risk of non‐specific staining of other cell populations. Translation of these research efforts into clinical immunophenotyping for diagnostic and prognostic applications will require the application of reproducible methods, along with clear reporting of excitation and emission values used, as well as greater focus on clinical and/or primary samples over immortalised and animal models.

## Conflicts of Interest

The authors declare no conflicts of interest.

## Data Availability

Data sharing not applicable to this article as no datasets were generated or analysed during the current study.

## References

[1] S. Akira , “Innate Immunity and Adjuvants,” Philosophical Transactions of the Royal Society, B: Biological Sciences 366, no. 1579 (2011): 2748–2755, 10.1098/rstb.2011.0106. PMC3146784 21893536

[2] J. Gao , Y. Luo , H. Li , et al., “Deep Immunophenotyping of Human Whole Blood by Standardized Multi‐Parametric Flow Cytometry Analyses,” Phenomics 3, no. 3 (2023): 309–328, 10.1007/s43657-022-00092-9. 37325713 PMC10260734

[3] Y.‐P. Chen , J.‐H. Yin , W.‐F. Li , et al., “Single‐Cell Transcriptomics Reveals Regulators Underlying Immune Cell Diversity and Immune Subtypes Associated With Prognosis in Nasopharyngeal Carcinoma,” Cell Research 30, no. 11 (2020): 1024–1042. 32686767 10.1038/s41422-020-0374-x PMC7784929

[4] D. Dyikanov , A. Zaitsev , T. Vasileva , et al., “Comprehensive Peripheral Blood Immunoprofiling Reveals Five Immunotypes With Immunotherapy Response Characteristics in Patients With cancer,” Cancer Cell 42, no. 5 (2024): 759–779, 10.1016/j.ccell.2024.04.008. 38744245

[5] N. Tchitchek , M. Binvignat , A. Roux , et al., “Deep Immunophenotyping Reveals That Autoimmune and Autoinflammatory Disorders Are Spread Along Two Immunological Axes Capturing Disease Inflammation Levels and Types,” Annals of the Rheumatic Diseases 83, no. 5 (2024): 638–650, 10.1136/ard-2023-225179. 38182406 PMC11041612

[6] J. M. Campbell , M. Gosnell , A. Agha , et al., “Label‐Free Assessment of Key Biological Autofluorophores: Material Characteristics and Opportunities for Clinical Applications,” Advanced Materials (Deerfield Beach, Fla.) 36, no. 42 (2024): 2403761, 10.1002/adma.202403761. 38775184

[7] M. E. Gosnell , A. G. Anwer , S. B. Mahbub , et al., “Quantitative Non‐invasive Cell Characterisation and Discrimination Based on Multispectral Autofluorescence Features,” Scientific Reports 6 (2016): 23453, 10.1038/srep23453. 27029742 PMC4814840

[8] S. B. Mahbub , A. Guller , J. M. Campbell , et al., “Non‐Invasive Monitoring of Functional State of Articular Cartilage Tissue With Label‐Free Unsupervised Hyperspectral Imaging,” Scientific Reports 9 (2019): 4398, 10.1038/s41598-019-40942-7. 30867549 PMC6416344

[9] B. Banerjee , T. Renkoski , L. R. Graves , et al., “Tryptophan Autofluorescence Imaging of Neoplasms of the Human colon,” Journal of Biomedical Optics 17, no. 1 (2012): 16003, 10.1117/1.JBO.17.1.016003. 22352653

[10] B. Banerjee , N. S. Rial , T. Renkoski , et al., “Enhanced Visibility of Colonic Neoplasms Using Formulaic Ratio Imaging of Native Fluorescence,” Lasers in Surgery and Medicine 45, no. 9 (2013): 573–581, 10.1002/lsm.22186. 24114774 PMC4040277

[11] L. S. Lin , F. W. Yang , and S. S. Xie , “Extracting Autofluorescence Spectral Features for Diagnosis of Nasopharyngeal Carcinoma,” Laser Physics 22, no. 9 (2012): 1431–1434, 10.1134/s1054660x12090095.

[12] J. M. Campbell , S. Mahbub , A. Habibalahi , S. Paton , S. Gronthos , and E. Goldys , “Ageing Human Bone Marrow Mesenchymal Stem Cells Have Depleted NAD (P) H and Distinct Multispectral Autofluorescence,” GeroScience 43 (2021): 859–868. 32789662 10.1007/s11357-020-00250-9 PMC8110641

[13] C. M. Chandrasekara , G. Gemikonakli , J. Mach , et al., “Ageing and Polypharmacy in Mesenchymal Stromal Cells: Metabolic Impact Assessed by Hyperspectral Imaging of Autofluorescence,” International Journal of Molecular Sciences 25, no. 11 (2024): 5830. 38892017 10.3390/ijms25115830 PMC11171960

[14] J. M. Campbell , A. Habibalahi , S. Mahbub , et al., “Non‐destructive, Label Free Identification of Cell Cycle Phase in cancer Cells by Multispectral Microscopy of Autofluorescence,” BMC Cancer 19 (2019): 1–11. 31864316 10.1186/s12885-019-6463-x PMC6925881

[15] A. Habibalahi , M. D. Moghari , J. M. Campbell , et al., “Non‐invasive Real‐Time Imaging of Reactive Oxygen Species (ROS) Using Auto‐Fluorescence Multispectral Imaging Technique: A Novel Tool for Redox Biology,” Redox Biology 34 (2020): 101561. 32526699 10.1016/j.redox.2020.101561 PMC7287272

[16] T. M. Heaster , A. J. Walsh , Y. Zhao , S. W. Hiebert , and M. C. Skala , “Autofluorescence Imaging Identifies Tumor Cell‐Cycle Status on a Single‐Cell Level,” Journal of Biophotonics 11, no. 1 (2018): e201600276, 10.1002/jbio.201600276. PMC5680147 28485124

[17] N. Pavillon , A. J. Hobro , S. Akira , and N. I. Smith , “Noninvasive Detection of Macrophage Activation With Single‐Cell Resolution Through Machine Learning,” Proceedings of the National Academy of Sciences of the United States of America 115, no. 12 (2018): e2676–e2685, 10.1073/pnas.1711872115. 29511099 PMC5866539

[18] D. E. Fuerst and J. R. Jannach , “Autofluorescence of Eosinophils: A Bone Marrow Study,” Nature 205, no. 4978 (1965): 1333–1334, 10.1038/2051333a0.

[19] S. M. Watt , A. W. Burgess , D. Metcalf , and F. L. Battye , “Isolation of Mouse Bone Marrow Neutrophils by Light Scatter and Autofluorescence,” Journal of Histochemistry and Cytochemistry 28, no. 9 (1980): 934–946, 10.1177/28.9.7410816. 7410816

[20] K. Lisy and K. Porritt , “Narrative Synthesis: Considerations and Challenges,” JBI Evidence Implementation 14, no. 4 (2016): 201, 10.1097/01.XEB.0000511348.97198.8c.

[21] I. J. Bauer , P. Fang , K. F. Lämmle , et al., “Visualizing the Activation of Encephalitogenic T Cells in the Ileal lamina Propria by In Vivo Two‐Photon Imaging,” Proceedings of the National Academy of Sciences 120, no. 30 (2023): e2302697120, https://www.ncbi.nlm.nih.gov/pmc/articles/PMC10372570/pdf/pnas.202302697.pdf. 10.1073/pnas.2302697120 PMC10372570 37467267

[22] A. Bullen , R. S. Friedman , and M. F. Krummel , “Two‐Photon Imaging of the Immune System: A Custom Technology Platform for High‐Speed, Multicolor Tissue Imaging of Immune Responses,” in Visualizing Immunity, eds. M. Dustin and D. McGavern , vol. 334 (Springer, 2009), 1–29. 10.1007/978-3-540-93864-4_1 19521679

[23] T. Chtanova , H. R. Hampton , L. A. Waterhouse , et al., “Real‐Time Interactive Two‐Photon Photoconversion of Recirculating Lymphocytes for Discontinuous Cell Tracking in Live Adult Mice,” Journal of Biophotonics 7, no. 6 (2014): 425–433. 23184395 10.1002/jbio.201200175

[24] P. Maffia , B. H. Zinselmeyer , A. Ialenti , et al., “Images in Cardiovascular Medicine: Multiphoton Microscopy for Three‐Dimensional Imaging of Lymphocyte Recruitment Into Apolipoprotein‐E‐Deficient Mouse Carotid Artery,” Circulation 115, no. 11 (2007): e326–e328. 17372180 10.1161/CIRCULATIONAHA.106.658492

[25] U. Maus , S. Rosseau , W. Seeger , and J. Lohmeyer , “Separation of Human Alveolar Macrophages by Flow Cytometry,” American Journal of Physiology 272, no. 3 Pt 1 (1997): L566–L571, 10.1152/ajplung.1997.272.3.L566. 9124615

[26] A. J. Mitchell , L. C. Pradel , L. Chasson , et al., “Technical Advance: Autofluorescence as a Tool for Myeloid Cell Analysis,” Journal of Leukocyte Biology 88, no. 3 (2010): 597–603, 10.1189/jlb.0310184. 20534703

[27] T.‐R. Riew , H. L. Kim , J.‐H. Choi , X. Jin , Y.‐J. Shin , and M.‐Y. Lee , “Progressive Accumulation of Autofluorescent Granules in Macrophages in Rat Striatum After Systemic 3‐Nitropropionic Acid: A Correlative Light‐ and electron‐Microscopic Study,” Histochemistry and Cell Biology 148, no. 5 (2017): 517–528, 10.1007/s00418-017-1589-x. 28597061

[28] C. M. Sköld , A. Eklund , G. Halldén , and J. Hed , “Autofluorescence in Human Alveolar Macrophages From Smokers: Relation to Cell Surface Markers and Phagocytosis,” Experimental Lung Research 15, no. 6 (1989): 823–835, 10.3109/01902148909069629. 2612443

[29] C. M. Sköld , A. Eklund , G. Halldén , and J. Hed , “Different Cell Surface and Phagocytic Properties in Mononuclear Phagocytes From Blood and Alveoli. A Comparative Study of Blood Monocytes and Alveolar Macrophages From Human Nonsmokers Using Flow Cytofluorometry,” APMIS 98, no. 5 (1990): 415–422, 10.1111/j.1699-0463.1990.tb01052.x. 2141477

[30] C. Tu , X. Ma , P. Pantazis , S. M. Kauzlarich , and A. Y. Louie , “Paramagnetic, Silicon Quantum Dots for Magnetic Resonance and Two‐Photon Imaging of Macrophages,” Journal of the American Chemical Society 132, no. 6 (2010): 2016–2023, https://www.ncbi.nlm.nih.gov/pmc/articles/PMC2836323/pdf/nihms‐172193.pdf. 20092250 10.1021/ja909303g PMC2836323

[31] J. James , D. Kantere , J. Enger , J. Siarov , A. M. Wennberg , and M. B. Ericson , “Report on Fluorescence Lifetime Imaging Using Multiphoton Laser Scanning Microscopy Targeting Sentinel Lymph Node Diagnostics,” Journal of Biomedical Optics 25, no. 7 (2020): 1–8, 10.1117/1.Jbo.25.7.071204. PMC7070082 32172545

[32] A. Klinger , R. Orzekowsky‐Schroeder , D. von Smolinski , et al., “Complex Morphology and Functional Dynamics of Vital Murine Intestinal Mucosa Revealed by Autofluorescence 2‐Photon Microscopy,” Histochemistry and Cell Biology 137, no. 3 (2012): 269–278, 10.1007/s00418-011-0905-0. 22227801 PMC3278620

[33] W. T. Kong , M. Bied , and F. Ginhoux , “Spectral Flow Cytometry Analysis of Resident Tissue Macrophages,” Methods in Molecular Biology 2713 (2024): 269–280, 10.1007/978-1-0716-3437-0_18. 37639129

[34] J. C. Lay , D. B. Peden , and N. E. Alexis , “Flow Cytometry of Sputum: Assessing Inflammation and Immune Response Elements in the Bronchial Airways,” Inhalation Toxicology 23, no. 7 (2011): 392–406, https://www.ncbi.nlm.nih.gov/pmc/articles/PMC3677049/pdf/nihms317901.pdf. 21639708 10.3109/08958378.2011.575568 PMC3677049

[35] S. Maiti , J. B. Shear , R. M. Williams , W. R. Zipfel , and W. W. Webb , “Measuring Serotonin Distribution in Live Cells With Three‐Photon Excitation,” Science 275, no. 5299 (1997): 530–532, 10.1126/science.275.5299.530. 8999797

[36] A. Maoz , C. Merenstein , Y. Koga , et al., “Elevated T Cell Repertoire Diversity Is Associated With Progression of Lung Squamous Cell Premalignant Lesions,” Journal for Immunotherapy of Cancer 9, no. 9 (2021): e002647, 10.1136/jitc-2021-002647. 34580161 PMC8477334

[37] N. Pavillon and N. I. Smith , “Immune Cell Type, Cell Activation, and Single Cell Heterogeneity Revealed by Label‐Free Optical Methods,” Scientific Reports 9, no. 1 (2019): 17054, https://www.nature.com/articles/s41598‐019‐53428‐3.pdf. 31745140 10.1038/s41598-019-53428-3 PMC6864054

[38] T. G. Phan and A. Bullen , “Practical Intravital Two‐Photon Microscopy for Immunological Research: Faster, Brighter, Deeper,” Immunology and Cell Biology 88, no. 4 (2010): 438–444. 20066001 10.1038/icb.2009.116

[39] A. B. Shrirao , R. S. Schloss , Z. Fritz , M. V. Shrirao , R. Rosen , and M. L. Yarmush , “Autofluorescence of Blood and Its Application in Biomedical and Clinical Research,” Biotechnology and Bioengineering 118, no. 12 (2021): 4550–4576. 34487351 10.1002/bit.27933

[40] A. R. Ambrose , S. Dechantsreiter , R. Shah , et al., “Corrected Super‐Resolution Microscopy Enables Nanoscale Imaging of Autofluorescent Lung Macrophages,” Biophysical Journal 119, no. 12 (2020): 2403–2417, 10.1016/j.bpj.2020.10.041. 33217385 PMC7822748

[41] M. Yadav , “Innate Immunity,” in An Interplay of Cellular and Molecular Components of Immunology (CRC Press, 2022), 27–59.

[42] T. P. Monie , The Innate Immune System: A Compositional and Functional Perspective (Academic Press, 2017).

[43] M. Monici , R. Pratesi , P. A. Bernabei , et al., “Natural Fluorescence of White Blood Cells: Spectroscopic and Imaging Study,” Journal of Photochemistry and Photobiology. B 30, no. 1 (1995): 29–37, 10.1016/1011-1344(95)07149-v. 8558361

[44] N. Safdarian , Z. Liu , T. D. Wang , and E. T. Wang , “Identification of Nasal Eosinophils Using Two‐Photon Excited Fluorescence,” Annals of Allergy, Asthma & Immunology 106, no. 5 (2011): 394–400, 10.1016/j.anai.2010.12.019. PMC4266399 21530871

[45] A. Klinder , J. Markhoff , A. Jonitz‐Heincke , P. Sterna , A. Salamon , and R. Bader , “Comparison of Different Cell Culture Plates for the Enrichment of Non‐adherent Human Mononuclear Cells,” Experimental and Therapeutic Medicine 17, no. 3 (2019): 2004–2012. 30867690 10.3892/etm.2019.7204 PMC6395970

[46] B. P. Yakimov , M. A. Gogoleva , A. N. Semenov , et al., “Label‐Free Characterization of White Blood Cells Using Fluorescence Lifetime Imaging and Flow‐Cytometry: Molecular Heterogeneity and Erythrophagocytosis [Invited],” Biomedical Optics Express 10, no. 8 (2019): 4220–4236, 10.1364/boe.10.004220. 31453006 PMC6701549

[47] Y. Adachi , A. L. Kindzelskii , A. R. Petty , et al., “IFN‐Gamma Primes RAW264 Macrophages and Human Monocytes for Enhanced Oxidant Production in Response to CpG DNA via Metabolic Signaling: Roles of TLR9 and Myeloperoxidase Trafficking,” Journal of Immunology 176, no. 8 (2006): 5033–5040, 10.4049/jimmunol.176.8.5033. 16585600

[48] T. N. Glenn , A. A. Oraevsky , F. K. Tittel , S. L. Jacques , and P. D. Henry , “Oxidatively Modified Low‐Density Lipoprotein in Mononuclear Cells Detected by Laser‐Induced Fluorescence Spectroscopy,” Proceedings of SPIE 2395 (1995): 368–375.

[49] T. N. Fink , A. A. Oraevsky , F. K. Tittel , S. L. Thomsen , and S. L. Jacques , “Autofluorescence Detection of Oxidized LDL in Monocytes: A Novel Risk Factor for the Assessment of Atherosclerosis? Advances in Laser and Light Spectroscopy to Diagnose Cancer and Other Diseases III: Optical Biopsy,” (1996).

[50] T. M. Heaster , M. Humayun , J. Yu , D. J. Beebe , and M. C. Skala , “Autofluorescence Imaging of 3D Tumor‐Macrophage Microscale Cultures Resolves Spatial and Temporal Dynamics of Macrophage Metabolism,” Cancer Research 80, no. 23 (2020): 5408–5423, 10.1158/0008-5472.Can-20-0831. 33093167 PMC7718391

[51] A. K. Abbas , A. H. Lichtman , S. Pillai , and D. H. Baker , Basic Immunology: Functions and Disorders of the Immune System, 6th ed. (Elsevier, 2020).

[52] G. Hoeffel and F. Ginhoux , “Fetal Monocytes and the Origins of Tissue‐Resident Macrophages,” Cellular Immunology 330 (2018): 5–15, 10.1016/j.cellimm.2018.01.001. 29475558

[53] J. M. Njoroge , L. B. Mitchell , M. Centola , D. Kastner , M. Raffeld , and J. L. Miller , “Characterization of Viable Autofluorescent Macrophages Among Cultured Peripheral Blood Mononuclear Cells,” Cytometry 44, no. 1 (2001): 38–44, 10.1002/1097-0320(20010501)44:1<38::aid-cyto1080>3.0.co;2-t. 11309807

[54] S. Lemire , O. M. Thoma , L. Kreiss , et al., “Natural NADH and FAD Autofluorescence as Label‐Free Biomarkers for Discriminating Subtypes and Functional States of Immune Cells,” International Journal of Molecular Sciences 23, no. 4 (2022): 2338, 10.3390/ijms23042338. 35216453 PMC8880312

[55] L. Lei , R. Tzekov , S. Tang , and S. Kaushal , “Accumulation and Autofluorescence of Phagocytized Rod Outer Segment Material in Macrophages and Microglial Cells,” Molecular Vision 18 (2012): 103–113, https://www.ncbi.nlm.nih.gov/pmc/articles/PMC3265176/pdf/mv‐v18‐103.pdf. 22275801 PMC3265176

[56] U. Gehlsen , M. Szaszák , A. Gebert , N. Koop , G. Hüttmann , and P. Steven , “Non‐Invasive Multi‐Dimensional Two‐Photon Microscopy Enables Optical Fingerprinting (TPOF) of Immune Cells,” Journal of Biophotonics 8, no. 6 (2015): 466–479, 10.1002/jbio.201400036. 25186637

[57] E. P. Kable and A. K. Kiemer , “Non‐invasive Live‐Cell Measurement of Changes in Macrophage NAD (P) H by Two‐Photon Microscopy,” Immunology Letters 96, no. 1 (2005): 33–38. 15585305 10.1016/j.imlet.2003.12.013

[58] C.‐T. Lin , E.‐K. Tien , S.‐Y. Lee , et al., “Effects of Ox‐LDL on Macrophages NAD(P)H Autofluorescence Changes by Two‐photon Microscopy,” Arxiv Preprint (2007) arXiv:0708.1849, 10.48550/arXiv.0708.1849.

[59] P. Bourdely , L. Petti , S. Khou , et al., “Autofluorescence Identifies Highly Phagocytic Tissue‐Resident Macrophages in Mouse and Human Skin and Cutaneous Squamous Cell Carcinoma,” Frontiers in Immunology 13 (2022): 903069, 10.3389/fimmu.2022.903069. 36325333 PMC9619110

[60] I. Smokelin , C. Mizzoni , J. Erndt‐Marino , D. Kaplan , and I. Georgakoudi , “Optical Changes in THP‐1 Macrophage Metabolism in Response to Pro‐ and Anti‐Inflammatory Stimuli Reported by Label‐Free Two‐Photon Imaging,” Journal of Biomedical Optics 25, no. 1 (2020): 1–14, 10.1117/1.Jbo.25.1.014512. PMC7008597 31953928

[61] A. Alfonso‐García , T. D. Smith , R. Datta , et al., “Label‐Free Identification of Macrophage Phenotype by Fluorescence Lifetime Imaging Microscopy,” Journal of Biomedical Optics 21, no. 4 (2016): 46005, https://www.ncbi.nlm.nih.gov/pmc/articles/PMC4833856/pdf/JBO‐021‐046005.pdf. 27086689 10.1117/1.JBO.21.4.046005 PMC4833856

[62] L. Q. Fu , W. L. Du , M. H. Cai , J. Y. Yao , Y. Y. Zhao , and X. Z. Mou , “The Roles of Tumor‐Associated Macrophages in Tumor Angiogenesis and Metastasis,” Cellular Immunology 353 (2020): 104119, 10.1016/j.cellimm.2020.104119. 32446032

[63] T. M. Heaster , A. R. Heaton , P. M. Sondel , and M. C. Skala , “Intravital Metabolic Autofluorescence Imaging Captures Macrophage Heterogeneity Across Normal and Cancerous Tissue,” Frontiers in Bioengineering and Biotechnology 9 (2021): 644648, 10.3389/fbioe.2021.644648. 33959597 PMC8093439

[64] J. M. Szulczewski , D. R. Inman , D. Entenberg , et al., “In Vivo Visualization of Stromal Macrophages via Label‐Free FLIM‐Based Metabolite Imaging,” Scientific Reports 6, no. 1 (2016): 25086. 27220760 10.1038/srep25086 PMC4879594

[65] M. Yang , A. Mahanty , C. Jin , A. N. N. Wong , and J. S. Yoo , “Label‐Free Metabolic Imaging for Sensitive and Robust Monitoring of Anti‐CD47 Immunotherapy Response in Triple‐Negative Breast cancer,” Journal for Immunotherapy of Cancer 10, no. 9 (2022): e005199, 10.1136/jitc-2022-005199. 36096527 PMC9472253

[66] L. Marcu , Q. Fang , J. A. Jo , et al., “In Vivo Detection of Macrophages in a Rabbit Atherosclerotic Model by Time‐Resolved Laser‐Induced Fluorescence Spectroscopy,” Atherosclerosis 181, no. 2 (2005): 295–303, 10.1016/j.atherosclerosis.2005.02.010. 16039283 PMC2672099

[67] J. J. Rico‐Jimenez , M. J. Serafino , S. Shrestha , et al., “Automated Detection of Superficial Macrophages in Atherosclerotic Plaques Using Autofluorescence Lifetime Imaging,” Atherosclerosis 285 (2019): 120–127, 10.1016/j.atherosclerosis.2019.04.223. 31051415 PMC6536321

[68] L. Chen , G. Qin , Y. Liu , et al., “Label‐Free Optical Metabolic Imaging of Adipose Tissues for Prediabetes Diagnosis,” Theranostics 13, no. 11 (2023): 3550, https://www.ncbi.nlm.nih.gov/pmc/articles/PMC10334843/pdf/thnov13p3550.pdf. 37441598 10.7150/thno.82697 PMC10334843

[69] V. Miskolci , K. E. Tweed , M. R. Lasarev , et al., “In Vivo Fluorescence Lifetime Imaging of Macrophage Intracellular Metabolism During Wound Responses in Zebrafish,” eLife 11 (2022): e66080, 10.7554/eLife.66080. 35200139 PMC8871371

[70] A. J. Walsh and M. C. Skala , “Optical Metabolic Imaging Quantifies Heterogeneous Cell Populations,” Biomedical Optics Express 6, no. 2 (2015): 559–573, 10.1364/BOE.6.000559. 25780745 PMC4354590

[71] N. G. B. Neto , S. A. O'Rourke , M. Zhang , H. K. Fitzgerald , A. Dunne , and M. G. Monaghan , “Non‐invasive Classification of Macrophage Polarisation by 2P‐FLIM and Machine Learning,” eLife 11 (2022): e77373, 10.7554/eLife.77373. 36254592 PMC9578711

[72] T. Hourani , A. Perez‐Gonzalez , K. Khoshmanesh , et al., “Label‐Free Macrophage Phenotype Classification Using Machine Learning Methods,” Scientific Reports 13, no. 1 (2023): 5202, 10.1038/s41598-023-32158-7. 36997576 PMC10061362

[73] B. Allard , A. Panariti , and J. G. Martin , “Alveolar Macrophages in the Resolution of Inflammation, Tissue Repair, and Tolerance to Infection,” Frontiers in Immunology 9 (2018): 1777, 10.3389/fimmu.2018.01777. 30108592 PMC6079255

[74] H. J. Bunn , G. Woltmann , and J. Grigg , “Applicability of Laser Scanning Cytometry to Study Paediatric Alveolar Macrophages,” European Respiratory Journal 20, no. 6 (2002): 1437–1443, 10.1183/09031936.02.00033502. 12503701

[75] C. M. Sköld , C. Barck , J. Lundahl , and A. Johansson , “Different Functional and Morphological Characteristics in a Nonadherent Subpopulation of Human Macrophages Recovered by Bronchoalveolar Lavage,” European Respiratory Journal 8, no. 10 (1995): 1719–1724, 10.1183/09031936.95.08101719. 8586128

[76] R. J. Streck , H. M. Jezewski , M. I. Rodriguez , et al., “A Method for Isolating Human Lung Macrophages and Observations of Fluorescent Phagocytes From the Lungs of Habitual Cigarette Smokers,” Journal of Immunological Methods 174, no. 1–2 (1994): 67–82, 10.1016/0022-1759(94)90011-6. 8083540

[77] T. Kerényi , B. Voss , G. Goeckenjan , and K. M. Müller , “Cellular Autofluorescent Pigment and Interstitial Fibrosis in Smoker's Lung,” Pathology, Research and Practice 188, no. 7 (1992): 925–930, 10.1016/s0344-0338(11)80253-0. 1448383

[78] J. L. Pauly , E. M. Allison , E. L. Hurley , C. E. Nwogu , P. K. Wallace , and G. M. Paszkiewicz , “Fluorescent Human Lung Macrophages Analyzed by Spectral Confocal Laser Scanning Microscopy and Multispectral Cytometry,” Microscopy Research and Technique 67, no. 2 (2005): 79–89, 10.1002/jemt.20191. 16037980

[79] C. Reiter , “Fluorescence Test to Identify Deep Smokers,” Forensic Science International 31, no. 1 (1986): 21–26, 10.1016/0379-0738(86)90068-x. 3721370

[80] L. P. Nicod , M. F. Lipscomb , G. B. Toews , and J. C. Weissler , “Separation of Potent and Poorly Functional Human Lung Accessory Cells Based on Autofluorescence,” Journal of Leukocyte Biology 45, no. 5 (1989): 458–465, 10.1002/jlb.45.5.458. 2523463

[81] E. C. Soethout , K. E. Müller , A. J. van den Belt , and V. P. Rutten , “Identification and Phenotyping of Leukocytes in Bovine Bronchoalveolar Lavage Fluid,” Clinical and Diagnostic Laboratory Immunology 11, no. 4 (2004): 795–798, 10.1128/cdli.11.4.795-798.2004. 15242961 PMC440617

[82] M. Y. Chang , J. E. Brune , M. Black , W. A. Altemeier , and C. W. Frevert , “Multi‐Compartmental Analysis of the Murine Pulmonary Immune Response by Spectral Flow Cytometry,” American Journal of Physiology. Lung Cellular and Molecular Physiology 325 (2023): L518–L535, 10.1152/ajplung.00317.2022. 37581225 PMC10639014

[83] A. Verghese , B. Franzus , and R. Stout , “Characterization of Bronchoalveolar Lavage Cells and Macrophage‐Derived Chemoattractant Activity in Pancreatic Elastase‐Induced Emphysema in Hamsters,” Experimental Lung Research 14, no. 6 (1988): 797–810, 10.3109/01902148809087845. 3061789

[84] A. Berndt and G. Müller , “Heterogeneity of Porcine Alveolar Macrophages in Experimental Pneumonia,” Veterinary Immunology and Immunopathology 57, no. 3–4 (1997): 279–287, 10.1016/s0165-2427(97)00009-3. 9261965

[85] V. J. Jameson , T. Luke , Y. Yan , et al., “Unlocking Autofluorescence in the Era of Full Spectrum Analysis: Implications for Immunophenotype Discovery Projects,” Cytometry. Part A 101, no. 11 (2022): 922–941, 10.1002/cyto.a.24555. PMC9519814 35349225

[86] L. M. G. van Huizen , M. Blokker , Y. Rip , et al., “Leukocyte Differentiation in Bronchoalveolar Lavage Fluids Using Higher Harmonic Generation Microscopy and Deep Learning,” PLoS One 18, no. 6 (2023): e0279525, 10.1371/journal.pone.0279525. 37368904 PMC10298778

[87] M. Colonna and O. Butovsky , “Microglia Function in the Central Nervous System During Health and Neurodegeneration,” Annual Review of Immunology 35 (2017): 441–468, 10.1146/annurev-immunol-051116-052358. PMC8167938 28226226

[88] R. M. Ritzel , Y. Li , Y. Jiao , et al., “Brain Injury Accelerates the Onset of a Reversible Age‐Related Microglial Phenotype Associated With Inflammatory Neurodegeneration,” Science Advances 9, no. 10 (2023): eadd1101, 10.1126/sciadv.add1101. 36888713 PMC9995070

[89] J. C. Burns , B. Cotleur , D. M. Walther , et al., “Differential Accumulation of Storage Bodies With Aging Defines Discrete Subsets of Microglia in the Healthy Brain,” eLife 9 (2020): e57495, 10.7554/eLife.57495. 32579115 PMC7367682

[90] A. Rotte and M. Bhandaru , “Dendritic Cells,” in Immunotherapy of Melanoma (Springer International Publishing, 2016), 143–166, 10.1007/978-3-319-48066-4_6.

[91] M. Krystel‐Whittemore , K. N. Dileepan , and J. G. Wood , “Mast Cell: A Multi‐Functional Master Cell [review],” Frontiers in Immunology 6 (2016): 620, 10.3389/fimmu.2015.00620. 26779180 PMC4701915

[92] I. Buchwalow , D. Atiakshin , V. Samoilova , W. Boecker , and M. Tiemann , “Identification of Autofluorescent Cells in Human Angioimmunoblastic T‐Cell Lymphoma,” Histochemistry and Cell Biology 149, no. 2 (2018): 169–177, 10.1007/s00418-017-1624-y. 29197996

[93] C. A. Rubio , “A Method for the Detection of Eosinophilic Granulocytes in Colonoscopic Biopsies From IBD Patients,” Pathology, Research and Practice 199, no. 3 (2003): 145–150, 10.1078/0344-0338-00367. 12812315

[94] R. M. Williams , J. B. Shear , W. R. Zipfel , S. Maiti , and W. W. Webb , “Mucosal Mast Cell Secretion Processes Imaged Using Three‐Photon Microscopy of 5‐Hydroxytryptamine Autofluorescence,” Biophysical Journal 76, no. 4 (1999): 1835–1846, https://www.ncbi.nlm.nih.gov/pmc/articles/PMC1300160/pdf/10096882.pdf. 10096882 10.1016/S0006-3495(99)77343-1 PMC1300160

[95] S. J. Lillard and E. S. Yeung , “Temporal and Spatial Monitoring of Exocytosis With Native Fluorescence Imaging Microscopy,” Journal of Neuroscience Methods 75, no. 1 (1997): 103–109, 10.1016/s0165-0270(97)00059-9. 9262151

[96] C. Li , R. K. Pastila , and C. P. Lin , “Imaging Immune Response of Skin Mast Cells In Vivo With Two‐Photon Microscopy,” Proceedings of SPIE 8207 (2012): 82070F1.

[97] B. Geering , C. Stoeckle , S. Conus , and H.‐U. Simon , “Living and Dying for Inflammation: Neutrophils, Eosinophils, Basophils,” Trends in Immunology 34, no. 8 (2013): 398–409, 10.1016/j.it.2013.04.002. 23665135

[98] Y. Zeng , B. Yan , Q. Sun , et al., “Label‐Free In Vivo Imaging of Human Leukocytes Using Two‐Photon Excited Endogenous Fluorescence,” Journal of Biomedical Optics 18, no. 4 (2013): 40504, 10.1117/1.JBO.18.4.040504. 23552632

[99] Y. Zeng , B. Yan , Q. Sun , et al., “Two‐Photon Excited Endogenous Fluorescence for Label‐Free In Vivo Imaging Ingestion of Disease‐Causing Bacteria by Human Leukocytes,” Proceedings of SPIE 8588 (2013): 85881L.

[100] C. Li , R. K. Pastila , C. Pitsillides , et al., “Imaging Leukocyte Trafficking In Vivo With Two‐Photon‐Excited Endogenous Tryptophan Fluorescence,” Optics Express 18, no. 2 (2010): 988–999, https://www.ncbi.nlm.nih.gov/pmc/articles/PMC3369551/pdf/oe‐18‐2‐988.pdf. 20173920 10.1364/OE.18.000988 PMC3369551

[101] D. L. Heintzelman , R. Lotan , and R. R. Richards‐Kortum , “Characterization of the Autofluorescence of Polymorphonuclear Leukocytes, Mononuclear Leukocytes and Cervical Epithelial cancer Cells for Improved Spectroscopic Discrimination of Inflammation From Dysplasia,” Photochemistry and Photobiology 71, no. 3 (2000): 327–332. 10732451

[102] R. Orzekowsky‐Schroeder , A. Klinger , S. Freidank , et al., “Probing the Immune and Healing Response of Murine Intestinal Mucosa by Time‐Lapse 2‐Photon Microscopy of Laser‐Induced Lesions With Real‐Time Dosimetry,” Biomedical Optics Express 5, no. 10 (2014): 3521–3540, 10.1364/boe.5.003521. 25360369 PMC4206321

[103] A. Klinger , L. Krapf , R. Orzekowsky‐Schroeder , N. Koop , A. Vogel , and G. Hüttmann , “Intravital Autofluorescence 2‐Photon Microscopy of Murine Intestinal Mucosa With Ultra‐Broadband Femtosecond Laser Pulse Excitation: Image Quality, Photodamage, and Inflammation,” Journal of Biomedical Optics 20, no. 11 (2015): 116001, 10.1117/1.Jbo.20.11.116001. 26524678

[104] B. Amulic , C. Cazalet , G. L. Hayes , K. D. Metzler , and A. Zychlinsky , “Neutrophil Function: From Mechanisms to Disease,” Annual Review of Immunology 30 (2012): 459–489, 10.1146/annurev-immunol-020711-074942. 22224774

[105] D. Barnes , S. Aggarwal , S. Thomsen , M. Fitzmaurice , and R. Richards‐Kortum , “A Characterization of the Fluorescent Properties of Circulating Human Eosinophils,” Photochemistry and Photobiology 58, no. 2 (1993): 297–303, 10.1111/j.1751-1097.1993.tb09565.x. 8415921

[106] D. A. Dorward , C. D. Lucas , A. L. Alessandri , et al., “Technical Advance: Autofluorescence‐Based Sorting: Rapid and Nonperturbing Isolation of Ultrapure Neutrophils to Determine Cytokine Production,” Journal of Leukocyte Biology 94, no. 1 (2013): 193–202, 10.1189/jlb.0113040. 23667167 PMC3685014

[107] G. J. Weil and T. M. Chused , “Eosinophil Autofluorescence and Its Use in Isolation and Analysis of Human Eosinophils Using Flow Microfluorometry,” Blood 57, no. 6 (1981): 1099–1104. 7225570

[108] Y. Zeng , J. Xu , D. Li , L. Li , Z. Wen , and J. Y. Qu , “In Vivo Cytometry Using Two‐Photon Autofluorescence Microscopy,” Biomedical Optics and 3D Imaging (2012): BSu3A.20.

[109] B. Liang and H. R. Petty , “Imaging Neutrophil Activation: Analysis of the Translocation and Utilization of NAD(P)H‐Associated Autofluorescence During Antibody‐Dependent Target Oxidation,” Journal of Cellular Physiology 152, no. 1 (1992): 145–156, 10.1002/jcp.1041520119. 1618916

[110] D. G. Hafeman , H. M. McConnell , J. W. Gray , and P. N. Dean , “Neutrophil Activation Monitored by Flow Cytometry: Stimulation by Phorbol Diester Is an All‐Or‐None Event,” Science 215, no. 4533 (1982): 673–675, 10.1126/science.6800035. 6800035

[111] J. El‐Benna , M. Hurtado‐Nedelec , V. Marzaioli , J.‐C. Marie , M.‐A. Gougerot‐Pocidalo , and P. M.‐C. Dang , “Priming of the Neutrophil Respiratory Burst: Role in Host Defense and Inflammation,” Immunological Reviews 273, no. 1 (2016): 180–193, 10.1111/imr.12447. 27558335

[112] A. Monsel , S. Lécart , A. Roquilly , A. Broquet , and C. Jacqueline , “Analysis of Autofluorescence in Polymorphonuclear Neutrophils: A New Tool for,” PLoS One 9, no. 3 (2014): e92564. 24658436 10.1371/journal.pone.0092564 PMC3962417

[113] S. Kretschmer , M. Pieper , G. Hüttmann , et al., “Autofluorescence Multiphoton Microscopy for Visualization of Tissue Morphology and Cellular Dynamics in Murine and Human Airways,” Laboratory Investigation 96, no. 8 (2016): 918–931, 10.1038/labinvest.2016.69. 27400364 PMC4972900

[114] A. N. Mayeno , K. J. Hamann , and G. J. Gleich , “Granule‐Associated Flavin Adenine Dinucleotide (FAD) is Responsible for Eosinophil Autofluorescence,” Journal of Leukocyte Biology 51, no. 2 (1992): 172–175, 10.1002/jlb.51.2.172. 1431554

[115] M. K. Samoszuk and F. P. Espinoza , “Deposition of Autofluorescent Eosinophil Granules in Pathologic Bone Marrow Biopsies,” Blood 70, no. 2 (1987): 597–599. 3607291

[116] S. M. Meinderts , P. A. Oldenborg , B. M. Beuger , et al., “Human and Murine Splenic Neutrophils are Potent Phagocytes of IgG‐Opsonized Red Blood Cells,” Blood Advances 1, no. 14 (2017): 875–886, 10.1182/bloodadvances.2017004671. 29296731 PMC5737592

[117] M. A. Cooper , M. Colonna , and W. M. Yokoyama , “Hidden Talents of Natural Killers: NK Cells in Innate and Adaptive Immunity,” EMBO Reports 10, no. 10 (2009): 1103–1110, 10.1038/embor.2009.203. 19730434 PMC2759738

[118] R. L. Schmitz , K. E. Tweed , P. Rehani , et al., “Autofluorescence Lifetime Imaging Classifies Human Lymphocyte Activation and Subtype,” bioRxiv (2023), 10.1101/2023.01.23.525260.

[119] B. Abós , C. Bailey , and C. Tafalla , “Adaptive Immunity,” in Principles of Fish Immunology: From Cells and Molecules to Host Protection, ed. K. Buchmann and C. J. Secombes (Springer International Publishing, 2022), 105–140, 10.1007/978-3-030-85420-1_3.

[120] M. H. F. Ottoni , M. G. D. Santos , V. G. Almeida , et al., “Background Autofluorescence Induced by Plant Extracts in Human Lymphocytes: A Flow Cytometric Analysis of a Critical bias,” Journal of Immunological Methods 468 (2019): 1–9, 10.1016/j.jim.2019.02.007. 30802448

[121] E. P. Hawkins , H. K. Hawkins , and D. Armstrong , “Lymphocyte Autofluorescence: A Screening Procedure for Neurodegenerative Diseases,” Pediatric Neurology 2, no. 3 (1986): 160–166, 10.1016/0887-8994(86)90010-x. 2854739

[122] P. Steven , J. Rupp , G. Hüttmann , et al., “Experimental Induction and Three‐Dimensional Two‐Photon Imaging of Conjunctiva‐Associated Lymphoid Tissue,” Investigative Ophthalmology & Visual Science 49, no. 4 (2008): 1512–1517, 10.1167/iovs.07-0809. 18385070

[123] L. Rigacci , R. Alterini , P. A. Bernabei , et al., “Multispectral Imaging Autofluorescence Microscopy for the Analysis of Lymph‐Node Tissues,” Photochemistry and Photobiology 71, no. 6 (2000): 737–742. 10857370 10.1562/0031-8655(2000)071<0737:miamft>2.0.co;2

[124] T. Fischer , A. Klinger , D. von Smolinski , et al., “High‐Resolution Imaging of Living Gut Mucosa: Lymphocyte Clusters Beneath Intestinal M Cells Are Highly Dynamic Structures,” Cell and Tissue Research 380, no. 3 (2020): 539–546, 10.1007/s00441-020-03167-z. 31970486

[125] P. Shen and S. Fillatreau , “Antibody‐Independent Functions of B Cells: A Focus on Cytokines,” Nature Reviews Immunology 15, no. 7 (2015): 441–451, 10.1038/nri3857. 26065586

[126] S. M. Pantanelli , Z. Li , R. Fariss , S. P. Mahesh , B. Liu , and R. B. Nussenblatt , “Differentiation of Malignant B‐Lymphoma Cells From Normal and Activated T‐Cell Populations by Their Intrinsic Autofluorescence,” Cancer Research 69, no. 11 (2009): 4911–4917, 10.1158/0008-5472.Can-08-2761. 19458079 PMC2735025

[127] A. J. Walsh , K. P. Mueller , K. Tweed , et al., “Classification of T‐Cell Activation via Autofluorescence Lifetime Imaging,” Nature Biomedical Engineering 5, no. 1 (2021): 77–88, 10.1038/s41551-020-0592-z. PMC7854821 32719514

[128] L. Hu , N. Wang , E. Cardona , and A. J. Walsh , “Fluorescence Intensity and Lifetime Redox Ratios Detect Metabolic Perturbations in T Cells,” Biomedical Optics Express 11, no. 10 (2020): 5674–5688, 10.1364/boe.401935. 33149978 PMC7587263

[129] L. M. Gerland , L. Genestier , S. Peyrol , et al., “Autolysosomes Accumulate During In Vitro CD8+ T‐Lymphocyte Aging and May Participate in Induced Death Sensitization of Senescent Cells,” Experimental Gerontology 39, no. 5 (2004): 789–800, 10.1016/j.exger.2004.01.013. 15130673

[130] A. V. Izosimova , M. V. Shirmanova , V. I. Shcheslavskiy , et al., “FLIM of NAD(P)H in Lymphatic Nodes Resolves T‐Cell Immune Response to the Tumor,” International Journal of Molecular Sciences 23, no. 24 (2022): 15829, 10.3390/ijms232415829. 36555468 PMC9779489

[131] A. Izosimova , A. Mozherov , M. Shirmanova , et al., “Fluorescence Lifetime Imaging of NAD (P) HT Cells Autofluorescence in the Lymphatic Nodes to Assess the Effectiveness of Anti‐CTLA‐4 Immunotherapy,” Современные технологии в медицине 15, no. 3 (2023): 5–15. 10.17691/stm2023.15.3.01 PMC10904361 38435479

[132] A. J. Walsh , R. S. Cook , M. E. Sanders , et al., “Quantitative Optical Imaging of Primary Tumor Organoid Metabolism Predicts Drug Response in Breast Cancer,” Cancer Research 74, no. 18 (2014): 5184–5194, 10.1158/0008-5472.Can-14-0663. 25100563 PMC4167558

[133] E. N. Cardona and A. J. Walsh , “Identification of Rare Cell Populations in Autofluorescence Lifetime Image Data,” Cytometry. Part A 101, no. 6 (2022): 497–506, 10.1002/cyto.a.24534. PMC9302681 35038211

[134] Z. J. Wang , A. J. Walsh , M. C. Skala , and A. Gitter , “Classifying T Cell Activity in Autofluorescence Intensity Images With Convolutional Neural Networks,” Journal of Biophotonics 13, no. 3 (2020): e201960050, https://www.ncbi.nlm.nih.gov/pmc/articles/PMC7065628/pdf/JBIO‐13‐e201960050.pdf. 31661592 10.1002/jbio.201960050 PMC7065628

[135] K. Samimi , E. C. Guzman , S. M. Trier , D. L. Pham , T. Qian , and M. C. Skala , “Time‐Domain Single Photon‐Excited Autofluorescence Lifetime for Label‐Free Detection of T Cell Activation,” Optics Letters 46, no. 9 (2021): 2168–2171, https://www.ncbi.nlm.nih.gov/pmc/articles/PMC8109150/pdf/nihms‐1695028.pdf. 33929445 10.1364/OL.422445 PMC8109150

[136] Y. Adachi , A. L. Kindzelskii , N. Ohno , T. Yadomae , and H. R. Petty , “Amplitude and Frequency Modulation of Metabolic Signals in Leukocytes: Synergistic Role of IFN‐Gamma in IL‐6‐ and IL‐2‐Mediated Cell Activation,” Journal of Immunology 163, no. 8 (1999): 4367–4374. 10510377

[137] P. Steven , F. Bock , G. Hüttmann , and C. Cursiefen , “Intravital Two‐Photon Microscopy of Immune Cell Dynamics in Corneal Lymphatic Vessels,” PLoS One 6, no. 10 (2011): e26253, 10.1371/journal.pone.0026253. 22028842 PMC3197633

[138] L. Kreiss , I. Ganzleben , A. Mühlberg , et al., “Label‐Free Analysis of Inflammatory Tissue Remodeling in Murine Lung Tissue Based on Multiphoton Microscopy, Raman Spectroscopy and Machine Learning,” Journal of Biophotonics 15, no. 9 (2022): e202200073, 10.1002/jbio.202200073. 35611635

[139] A. R. Heaton , P. R. Rehani , A. Hoefges , et al., “Single Cell Metabolic Imaging of Tumor and Immune Cells In Vivo in Melanoma Bearing Mice,” Frontiers in Oncology 13 (2023): 1110503, 10.3389/fonc.2023.1110503. 37020875 PMC10067577

[140] C. Schwartz and D. Voehringer , “Identification of Murine Basophils by Flow Cytometry and Histology,” in Basophils and Mast Cells: Methods and Protocols, ed. B. F. Gibbs and F. H. Falcone (Springer, 2014), 229–237, 10.1007/978-1-4939-1173-8_17. 25149496

[141] R. Chen , J. Y. Chen , and L. W. Zhou , “Metabolic Patterns (NAD(P)H) in Rat Basophilic Leukemia (RBL‐2H3) Cells and Human Hepatocellular Carcinoma (Hep G2) Cells With Autofluorescence Imaging,” Ultrastructural Pathology 32, no. 5 (2008): 193–198, 10.1080/01913120802397752. 18958792

[142] K. Miyake , J. Ito , and H. Karasuyama , “Role of Basophils in a Broad Spectrum of Disorders [Review],” Frontiers in Immunology 13 (2022): 902494, 10.3389/fimmu.2022.902494. 35693800 PMC9186123

[143] S. Dutt , I. Hamza , and T. B. Bartnikas , “Molecular Mechanisms of Iron and Heme Metabolism,” Annual Review of Nutrition 42 (2022): 311–335, 10.1146/annurev-nutr-062320-112625. PMC9398995 35508203

[144] S.‐K. Seo and B. Kwon , “Immune Regulation Through Tryptophan Metabolism,” Experimental & Molecular Medicine 55, no. 7 (2023): 1371–1379, 10.1038/s12276-023-01028-7. 37394584 PMC10394086

[145] L. M. G. van Huizen , T. Radonic , F. van Mourik , et al., “Compact Portable Multiphoton Microscopy Reveals Histopathological Hallmarks of Unprocessed Lung Tumor Tissue in Real Time,” Translational Biophotonics 2, no. 4 (2020): e202000009, 10.1002/tbio.202000009. 34341777 PMC8311669

[146] J. M. Campbell , S. B. Mahbub , M. J. Bertoldo , et al., “Multispectral Autofluorescence Characteristics of Reproductive Aging in Old and Young Mouse Oocytes,” Biogerontology 23, no. 2 (2022): 237–249. 35211812 10.1007/s10522-022-09957-y PMC9023381

[147] A. Knab , A. G. Anwer , B. Pedersen , et al., “Towards Label‐Free Non‐invasive Autofluorescence Multispectral Imaging for Melanoma Diagnosis,” Journal of Biophotonics 17, no. 4 (2024): e202300402, 10.1002/jbio.202300402. 38247053

[148] Cytek , “Cytek® Amnis® ImageStream®X Mk II Imaging Flow Cytometer,” (2024), retrieved Oct 7, 2024, https://cytekbio.com/pages/imagestream.

[149] Revvity , “Operetta CLS High‐Content Analysis System,” (2024), retrieved December 23, 2024, https://www.revvity.com/product/operetta‐cls‐system‐hh16000020.

[150] M. J. Page , J. E. McKenzie , P. M. Bossuyt , et al., “The PRISMA 2020 Statement: An Updated Guideline for Reporting Systematic Reviews,” BMJ 372 (2021): n71, 10.1136/bmj.n71. 33782057 PMC8005924

